# NIR/pH-triggered aptamer-functionalized DNA origami nanovehicle for imaging-guided chemo-phototherapy

**DOI:** 10.1186/s12951-023-01953-9

**Published:** 2023-06-10

**Authors:** Mengyue Li, Geng Yang, Yue Zheng, Jiazhen Lv, Wanyi Zhou, Hanxi Zhang, Fengming You, Chunhui Wu, Hong Yang, Yiyao Liu

**Affiliations:** 1 Department of Pharmacy, Personalized Drug Therapy Key Laboratory of Sichuan Province, Sichuan Provincial People’s Hospital, School of Life Science and Technology, University of Electronic Science and Technology of China, Chengdu, P.R. China; 2 TCM Regulating Metabolic Diseases Key Laboratory of Sichuan Province, Hospital of Chengdu, University of Traditional Chinese Medicine, No. 39 Shi-er-qiao Road, Chengdu, Sichuan 610072 P.R. China

**Keywords:** DNA origami, AS1411 aptamer, Chemotherapy, Photothermal therapy, Imaging-guided therapy

## Abstract

Targeted chemo-phototherapy has received widespread attention in cancer treatment for its advantages in reducing the side effects of chemotherapeutics and improving therapeutic effects. However, safe and efficient targeted-delivery of therapeutic agents remains a major obstacle. Herein, we successfully constructed an AS1411-functionalized triangle DNA origami (TOA) to codeliver chemotherapeutic drug (doxorubicin, DOX) and a photosensitizer (indocyanine green, ICG), denoted as TOADI (DOX/ICG-loaded TOA), for targeted synergistic chemo-phototherapy. In vitro studies show that AS1411 as an aptamer of nucleolin efficiently enhances the nanocarrier’s endocytosis more than 3 times by tumor cells highly expressing nucleolin. Subsequently, TOADI controllably releases the DOX into the nucleus through the photothermal effect of ICG triggered by near-infrared (NIR) laser irradiation, and the acidic environment of lysosomes/endosomes facilitates the release. The downregulated Bcl-2 and upregulated Bax, Cyt c, and cleaved caspase-3 indicate that the synergistic chemo-phototherapeutic effect of TOADI induces the apoptosis of 4T1 cells, causing ~ 80% cell death. In 4T1 tumor-bearing mice, TOADI exhibits 2.5-fold targeted accumulation in tumor region than TODI without AS1411, and 4-fold higher than free ICG, demonstrating its excellent tumor targeting ability in vivo. With the synergetic treatment of DOX and ICG, TOADI shows a significant therapeutic effect of ~ 90% inhibition of tumor growth with negligible systemic toxicity. In addition, TOADI presents outstanding superiority in fluorescence and photothermal imaging. Taken together, this multifunctional DNA origami-based nanosystem with the advantages of specific tumor targeting and controllable drug release provides a new strategy for enhanced cancer therapy.

**Supplementary Information:**

The online version contains supplementary material available at 10.1186/s12951-023-01953-9.

## Introduction

Chemotherapy is a typical treatment of cancer in clinics using antineoplastic drugs. For example, doxorubicin (DOX) is one of the most potent chemotherapeutic drugs approved by the Food and Drug Administration (FDA) [1]. However, the application of chemotherapeutic drugs is severely restricted by adverse effects such as cardiotoxicity, myelotoxicity, and poor selectivity between tumors and normal cells [2]. To overcome these problems and improve therapeutic effects, targeted synergistic therapy is regarded as an attractive option. Photothermal therapy (PTT) is a noninvasive and local treatment that causes cancer cell death by converting light energy into heat from photothermal agents (PTAs) under laser irradiation [3, 4]. As a near-infrared (NIR) contrast agent approved by FDA [5], indocyanine green (ICG) has been widely used in NIR imaging-guided PTT because of its strong NIR absorption and excellent photothermal conversion efficiency [6]. Whereas, the disadvantages of instability in aqueous solution, poor photostability, and rapid elimination from the body limit its application [7, 8]. The combination of chemotherapy and PTT is gradually rising as a promising strategy for cancer treatment to enhance therapeutic efficacy, minimize side effects, and maintain a long-term prognosis [9, 10]. Although a variety of codelivery systems have been developed, for example, hollow mesoporous silica [11, 12], liposomes [13], and monolayer nanosheets [14], the multifunctional platform for actively targeted delivery of bioactive agents safely and efficiently remains a major obstacle.

DNA nanomaterial, which has received growing attention in recent years, possesses tremendous potential in biomedical applications owing to its advantages of excellent biocompatibility, customized nanoscale shapes, and precise spatial addressability [15–17]. First of all, DNA as the essential biological molecule in living organisms is generally non-toxic and biodegradable. The interaction of DNA nanomaterial and biological systems would not cause significant harm or adverse effects [18]. Secondly, the self-assembly of DNA nanomaterials is based on the principle of complementary base pairing. Therefore, the flexible programmability of building blocks fabricates a wide range of DNA architectures with precise and controllable shapes and sizes [19–21]. Thirdly, the addressability of DNA nanomaterial allows for the precise placement of functional groups, molecules, or nanoparticles at specific locations within the DNA nanostructure [22, 23]. It enables the construction of multi-functional drug delivery systems for tumor-targeting delivery and therapy.

The invention of DNA origami technology is a milestone of the development of DNA nanomaterials [24]. A long single-stranded DNA, for example, the M13mp18 phage genome as a scaffold strand is paired with hundreds of synthetic oligonucleotides as staple stands to self-assemble arbitrary 2D and 3D nanostructures [24, 25]. Therefore, DNA origami has incomparable superiority to deliver various oligonucleotides [26–29]. For instance, AS1411, as a nucleic acid aptamer of nucleolin highly expressed in breast cancer cells [30–33], has been incorporated into DNA nanostructures as a cancer-targeting ligand as well as a therapeutic agent [34–36]. DNA origami itself is capable of penetrating physiological barriers and membranes, then accumulating in tumor tissue by the enhanced permeability and retention (EPR) effect [37]. Meanwhile, AS1411 aptamer promotes cell internalization by nucleolin-mediated micropinocytosis [38]. Previous works have validated that DNA nanocarriers with AS1411 exhibit remarkably enhanced targeting and intracellular uptake to cancer cells [39, 40]. In addition, DNA origami is able to load various antitumor drugs. DOX as a classic anthracycline antibiotic can intercalate into the molecules between the planar bases of DNA [41, 42]. Pan et al. have found that loading DOX on the targeted DNA nanocarriers can effectively reduce its side effects [29]. Although there are few reports of loading ICG on DNA origami so far, it has been shown that DOX and ICG are coupled during the immobilization process [14, 43]. With these outstanding properties, multifunctional DNA origami is a promising candidate for the targeted co-delivery of chemotherapeutic drugs and photosensitizers into tumor cells for synergistic chemo-phototherapy.

**Scheme 1:**
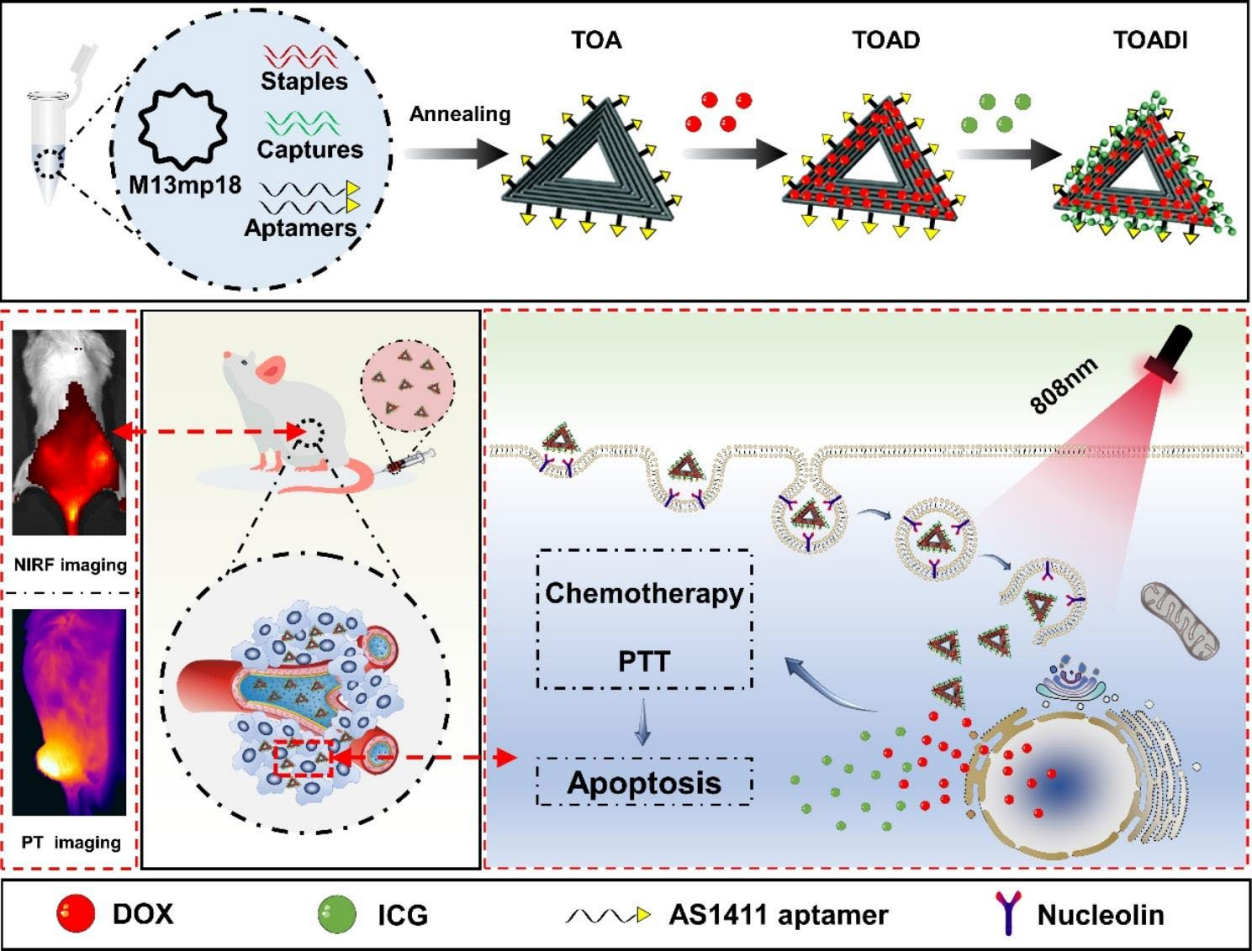
Schematic illustration showing the DNA origami-based nanoplatforms for synergistic cancer therapy involving PTT and chemotherapy against breast cancer in vitro and in vivo (TOA: Triangle DNA origami with AS1411; TOAD: Triangle DNA origami loaded with DOX; TOADI: Triangle DNA origami loaded with DOX and the ICG).

Herein, we describe a facile and common strategy to fabricate a DNA origami-based drugs codelivery nanovehicle to realize the combination of dual-mode imaging-guided PTT and chemotherapy (Scheme 1). A biocompatible triangle DNA origami (TO) with uniform size and shape was chosen to efficiently load DOX and ICG. To increase the targeting ability of DNA origami, AS1411 aptamer was utilized to construct AS1411 functionalized triangle DNA origami (TOA). Then, DOX was first loaded on TOA by intercalating into the tetracene ring system between the planar base pairs of duplex DNAs to form DOX-loaded TOA (TOAD). The loading of positive charged DOX converts negative charged DNA into positive. Finally, the negatively charged ICG was loaded on positively charged TOAD by electrostatic adsorption to form DOX/ICG-loaded TOA (TOADI). We demonstrated that TOADI can specifically target tumor cells highly expressing nucleolin. The release of DOX into the nucleus can be controlled by NIR laser irradiation, and the acidic environment facilitates the release. Further studies showed that TOADI induces the apoptosis of tumor cells, resulting in enhanced chemo-phototherapy for breast cancer. Besides, TOADI exhibits outstanding superiority in fluorescence and photothermal imaging for imaging-guided precise photothermal therapy. Taken together, this multifunctional DNA nanosystem combining PTT and chemotherapy exhibits a superior anticancer effect against 4T1 tumors in vitro and in vivo. To the best of our knowledge, it is the first report of the codelivery of DOX and ICG by DNA origami.

## Materials and methods

### Materials

All the chemicals and solvents were used at the analytical level. All aqueous solutions were prepared using millipore water. All oligonucleotides were purchased from Sangon Biotech (China). The unmodified synthetic DNA oligos were purified by High Affinity Purification, while high-performance liquid chromatography was required to purify the modified DNA strands. M13mp18 single-stranded DNA (N4040S) was ordered from New England Biolabs (USA). To simplify experiments, DNA strands were all first normalized to 100 µM. Doxorubicin (DOX) hydrochloride was ordered from Shanghai Aladdin Biochemical Technology Co., Ltd. (China). ICG was purchased from Ruixibio (China). The Amicon Ultra Centrifugal Filters (MWCO-100 K) were ordered from Merck Millipore. Mouse 4T1 breast cancer cell line was ordered from American Type Culture Collection (ATCC, Manassas, VA, USA). The primary anti-Bax, anti-Bcl-2, and anti-cleaved caspase-3 mAb was ordered from Cell Signaling Technology (USA) and the primary anti-actin mAb was ordered from Beyotime (China). Horseradish peroxidase-conjugated secondary antibody was purchased from Shanghai Epizyme Biomedical Technology Co., Ltd (China).

### Preparation of triangular DNA origami

The initial triangular DNA origami was assembled according to Rothemund’s and Ding’s methods [24, 41]. The molar ratio of the long ssDNA scaffold (M13mp18, 7249 nt, 5 nM), helper strands (staple strands and capture strands for AS1411 aptamers loading, 30 nM), and AS1411 aptamers (60 nM) were 1:6:12. They were self-assembled in 1× TAE/Mg^2+^ buffer (40 mM Tris, 20 mM acetic acid, 2 mM EDTA and 12.5 mM magnesium acetate, pH 8.3) by slowly cooling from 95℃ to 25℃ by PCR program over 10 h. After that, the additional strands were filtered by 100 kDa MWCO centrifuge filters three times. Then PBS was used to elute the sample of DNA origami in filters to form a stock solution (1 mM doxorubicin, 0.7 mM ICG, and ∼20 nM origami). All DNA origami related samples were stored at 4℃.

### Characterizations of unloaded, DOX-loaded and DOX/ICG-loaded DNA origami

The original DNA origami, DOX-loaded and the DOX/ICG-loaded DNA origami were characterized by atomic force microscopy (AFM), UV-vis absorption and agarose gel electrophoresis (AGE). Before the imaging of AFM tapping-in-buffer mode was performed that 10 µL of the sample was spotted on a freshly cleaved mica surface. After 10 min adsorbing, the sample was subsequently washed three times by ddH_2_O, Then, the samples were air-dried at room temperature (RT) before imaging in ScanAsyst mode. 1% AGE staining was performed by premixed GelRed (10000:1). The constant voltage of 100 V for 40 min in 1× TAE-Mg^2+^ buffer was used to run gels, and the imaging was performed by a gel imaging system (Bio-rad, USA).

### Western blot analysis

Expression of the apoptosis-related proteins in 4T1 cells was detected by Western blot. Briefly, 4T1 cells were first seeded in 6-well plates and then incubated with phosphate buffered saline (PBS), DOX, TOAD, TOADI, and TOADI + L at a final triangular DNA origami concentration of 0.4 nM (corresponding to DOX: 20 µM, ICG: 14 µM) in 1 mL RPIM 1640 complete culture medium for 12 h. In the TOADI+L group, “+L” means cells were treated with 808 nm laser irradiation (1.0 W/cm^2^, 5 min) at 6 h. Then, PBS was used to wash the cells three times and the whole cell protein extraction was lysed by RIPA lysis buffer (Beyotime, Jiangsu, China), and the protein concentration was measured by the BCA protein assay kit (Beyotime, Jiangsu, China). Subsequently, various protein groups were isolated by 12.5% SDS-PAGE and moved to PVDF membranes (Millipore, Bedford, MA, USA) by a wet transfer cell (Bio-Rad). PVDF membranes were blocked at RT for 3 h in Tris buffered saline tween buffer (TBST) and then incubated with the primary antibodies rabbit anti-mouse Bax, Bcl-2, cleaved caspase-3 (Cell Signaling Technology, USA) and β-actin (Beyotime, China) overnight at 4℃. After the membranes were washed to eliminate non-bound primary antibodies, then with the HRP-labeled secondary antibody (Shanghai Epizyme Biomedical Technology Co., Ltd, China) at a 1:10000 dilution at RT for 2 h. At last, membranes were washed with TBST three times, and the chemiluminescence and fluorescence imaging system observed immunoreactive signals (Sagecreation, Beijing, China). ImageJ software was used to quantify the various protein expression.

### In vivo fluorescence imaging

To assess the biodistribution of triangular DNA origami with or without targeting aptamer, 4T1 tumor-bearing mice were divided into three groups randomly (n = 3 per group). Then, subcutaneous injection of 4T1 cells (2 × 10^6^ per mouse) into the right flank of female BALB/c mice was performed. Due to the strong absorbance of ICG at 808 nm in vivo imaging system, the mice were treated with equivalent doses (calculated by the ICG) of free ICG, TODI (-aptamer), or TOADI (+ aptamer) via tail vein injection in a 100 µL injection volume. Fluorescence imaging was done by Lumina Series III imaging (PerkinElmer, Inc., Waltham, MA, USA) at 2, 4, 8, and 24 h after injection. Twenty-four hours after administration, to perform ex vivo fluorescence imaging, the mice were sacrificed to collect the tumor tissues and major organs (PerkinElmer, Inc., Waltham, MA, USA).

### Animals and tumor model

Female BALB/c mice (17-19 g, 5 ~ 6 weeks) were ordered from Chengdu Yaokang Biotechnology Co., Ltd (Chengdu, China). Animals were subsequently treated after 4T1 cell inoculation when the tumors reached a volume of approximately 100 mm^3^ (volume = length × width^2^/2). The 4T1 tumor-bearing mice model was established by injecting approximately 2 × 10^5^ 4T1 cells in the right flank of female BALB/c mice.

### Tumor growth inhibition

When the volume of 4T1 tumors was around 100 mm^3^, mice were randomly divided into 6 groups (n = 5 per group). The mice were treated with equivalent doses (triangular DNA origami: 0.5 mg/kg, DOX: 3.0 mg/kg, and ICG: 2.8 mg/kg) of 0.9% saline, TOA, DOX, TOAD, TOADI, and TOADI+L via tail vein injection in a 100 µL injection volume every 6 days for 2 treatments. In the TOADI+L group, “+L” means tumors were treated with 808 nm laser irradiation (1.0 W/cm^2^, 5 min) at 8 h. During 14 days of treatment, the tumor size of each mouse was measured that the digital vernier caliper was used to quantify the longest (a) and shortest (b) diameters and the volume of the tumor was computed through V = 0.5ab^2^. And the weight of each mouse was weighed using an electronic balance. After 14 days of treatment, all the mice were euthanatized, and the tumor tissues were imaged and weighed. Ex vivo organs were performed H&E (hematoxylin and eosin) staining for histopathological evaluations.

### Statistical analysis

Statistical differences were evaluated by one-way analysis of variance (ANOVA) followed by Tukey multiple comparisons. Statistical analysis was conducted using Prism 8.3 (GraphPad, San Diego, CA, USA). A value of *p* < 0.05 was considered statistically significant.

## Results and discussion

### Preparation and characterization of DNA origami-based codelivery nanosystem

**Fig. 1:**
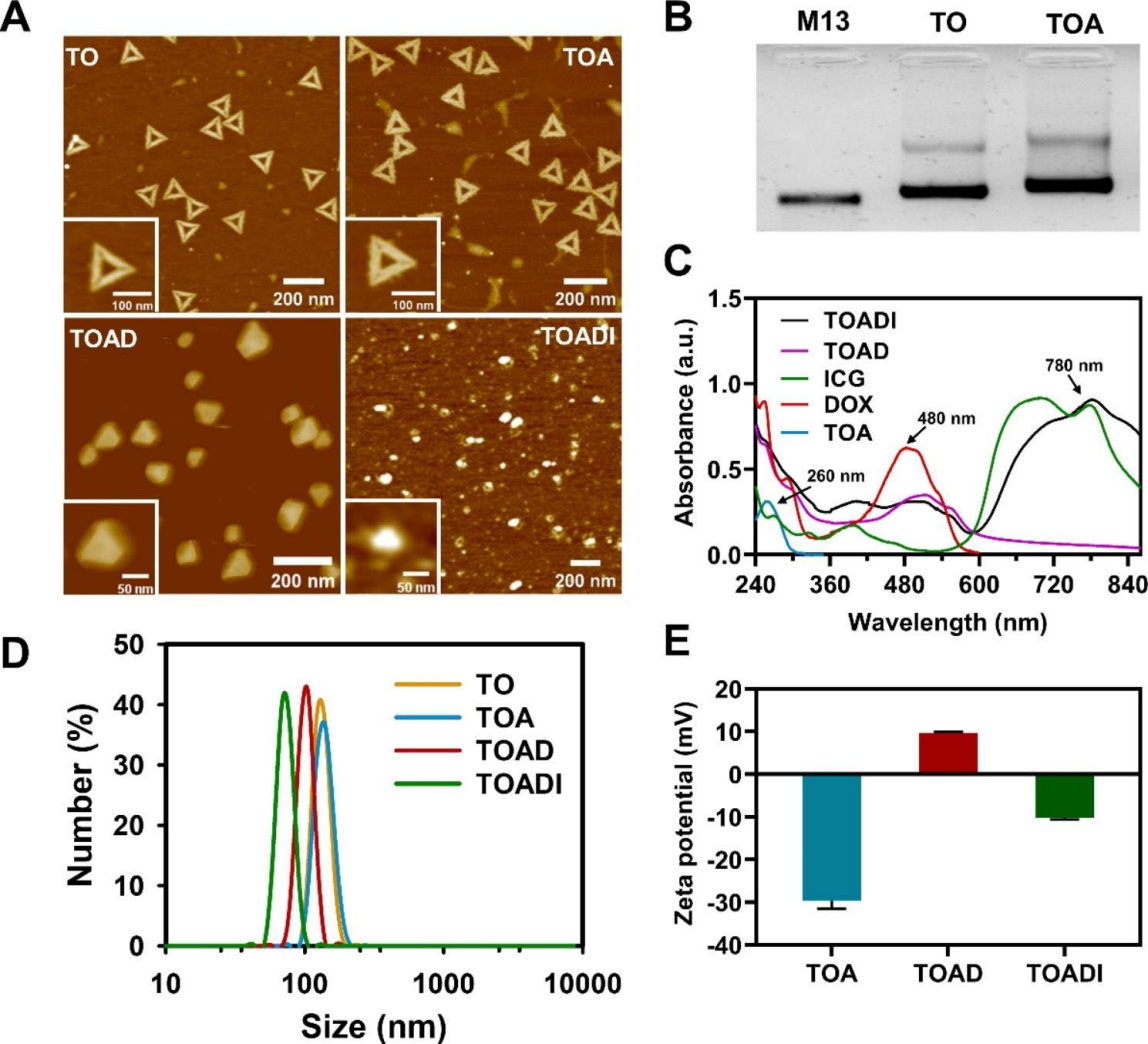
Characterizations of DNA-based nanocarrier. (**A**) AFM characterization of the sequential constructs in the generation of the targeted DNA origami loaded with DOX and ICG (TOADI). Insets: enlarged images. (**B**) 1% agarose gel electrophoresis analysis of the self-assembled triangle DNA origami with AS1411; lanes 1-3: M13 scaffold, TO and TOA, respectively. (**C**) UV-vis-NIR spectra of TOA, DOX, ICG, TOAD, and TOADI, respectively. (**D**) Dynamic light scattering of TO, TOA, TOAD, and TOADI. (**E**) Zeta-potential analysis of TOA, TOAD, and TOADI. (TO: Triangle DNA origami; TOA: Triangle DNA origami with AS1411; TOAD: Triangle DNA origami loaded with DOX; TOADI: Triangle DNA origami loaded with DOX and ICG). The data are presented as the mean ± SD (n = 3)

We first designed a facile strategy to fabricate a DNA origami-based nanovehicle for targeted codelivery of DOX and ICG. The triangle DNA origami was self-assembled by annealing the single-stranded M13mp18 and 208 short staples in an Eppendorf thermocycler from 95 to 25℃ over 10 h. The optimal ratio of M13mp18 to staples for the assembly of TO is 1:4-1:10 (**Figure **S1). To construct the AS1411 aptamer-functionalized DNA origami, 15 staples were replaced by corresponding modified staples with 5’-end extended AS1411 aptamer (**Scheme S1** and **Figure **S2). Then DNA origami was purified by centrifuging with 100 kDa Amicon ultrafilters and characterized by atomic force microscope (AFM) and 1% agarose gel electrophoresis. Trichomes-like spines at the edges of triangles and slower migration of TOA than that of TO both indicated AS1411 aptamers were successfully assembled on the DNA origami (Fig. 1A&**B**). Next, the chemotherapeutic drug DOX was loaded by intercalating into the base pairs of DNA duplex to form the DOX-loaded TOA. At last, the photosensitizer ICG was loaded through electrostatic interaction to form DOX/ICG-loaded TOA (Fig. 1A). The appearance characteristics of TOA, TOAD, and TOADI are shown in **Figure S3**. The UV-vis-NIR spectra showed that TOADI had the absorption peaks of DOX and ICG, suggesting successful loading (Fig. 1C). From the AFM images, we found that the loading of DOX and ICG caused the compaction of DNA origami (Fig. 1A). The dynamic light scattering assays showed that the size of the nanosystem was decreasing with the loading of DOX and ICG (TO: ~122 nm, TOA: ~136 nm, TOAD: ~106 nm, TOADI: ~72 nm) (Fig. 1D). This may be mainly because of electrostatic interaction. DNA is negatively charged, while the intercalated DOX is positively charged. The electrostatic attraction between DNA and DOX led to the first compaction of DNA origami, meanwhile, causing the positively charged TOAD (Fig. 1E). Then the electrostatic adsorption of negatively charged ICG caused the second compaction making the size of the nanosystem smaller.

### Drug release and photothermal effect evaluation

**Fig. 2:**
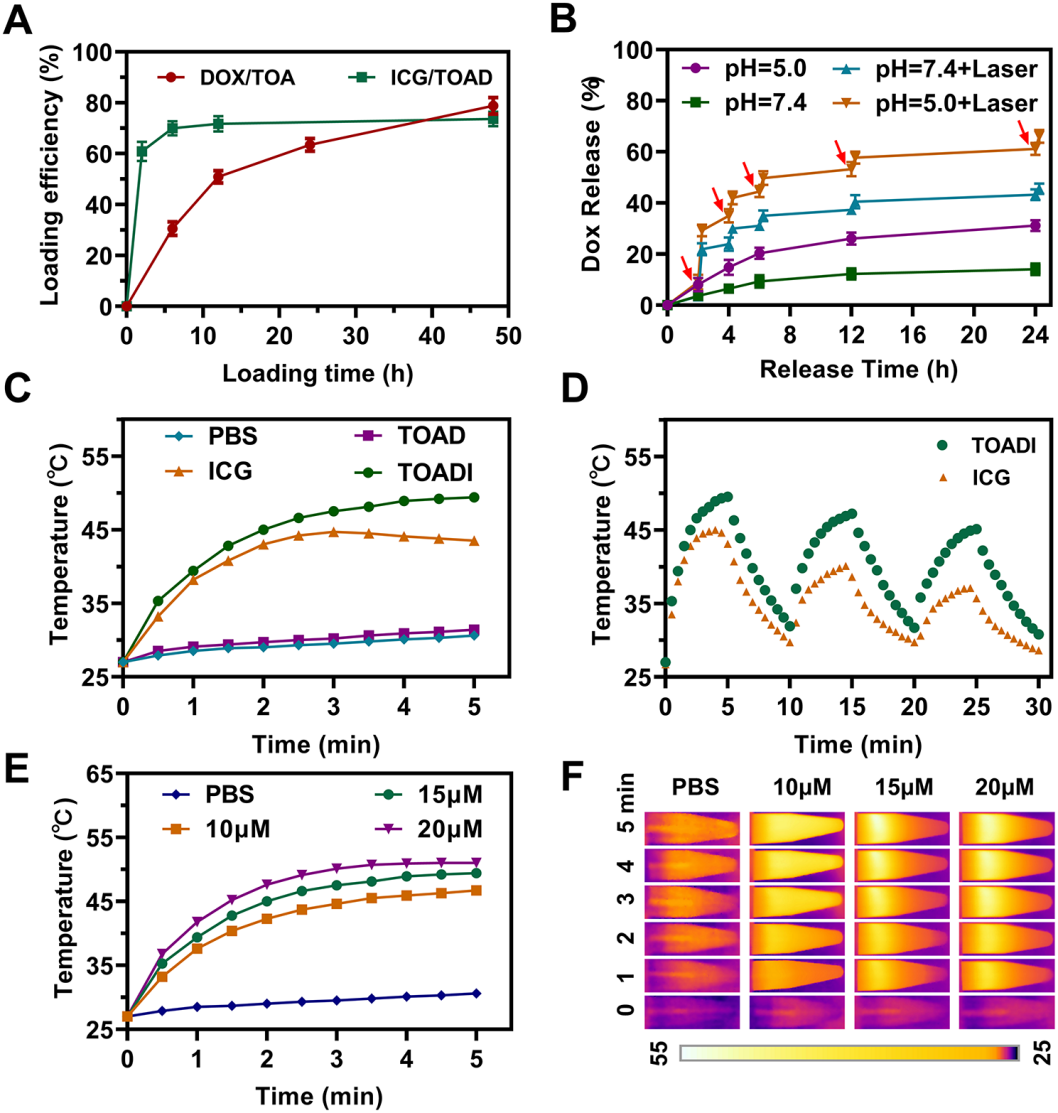
Loading efficiency and release of DOX and photothermal properties of DNA nanodevice. (**A**) Time-dependence of loading efficiency of DNA origami (20 nM) to DOX (2 mM) and ICG (1 mM). (**B**) DOX release profiles of TOADI at different pH values in the presence of laser irradiation (808 nm, 1.0 W/cm^2^, 5 min) at various time points. (**C**) The photothermal effect of TOADI (corresponding to DOX: 20 µM, ICG: 14 µM) under 808 nm irradiation (1.0 W/cm^2^), with ICG (14 µM), TOAD (corresponding to DOX: 20 µM) and PBS used as control samples. (**D**) Temperature evolution of ICG and TOADI dissolved in aqueous solution (ICG:14 µM) under 808 nm laser irradiation (1.0 W/cm^2^) after laser on/off cycles. (**E**) Temperature changes of TOADI dispersed in aqueous solution with various concentrations under 808 nm laser irradiation (1.0 W/cm^2^, 5 min). (**F**) Infrared thermal images at different concentrations and laser irradiation (808 nm, 1.0 W/cm^2^) were monitored by an infrared thermal camera in vitro. The data are presented as the mean ± SD (n = 3)

Next, the property of drug loading and release of TOADI was investigated. 2 mM DOX was incubated with 20 nM TOA to evaluate the loading efficiency of DOX on TOA. Over 50% of DOX was successfully loaded after 12 h incubation, and the loading efficiency of DOX was time-dependent (Fig. 2A and **Table **S1). Then 1 mM ICG was incubated with 20 nM TOAD, showing that the loading efficiency of ICG was over 70% and the loading of ICG reached the plateau region after 6 h incubation (Fig. 2A and **Table **S2). Subsequently, the efficiency of DOX release was assessed at pH 7.4 (approximate the blood pH value) and pH 5.0 (approximate the acidic endosomes/lysosomes pH value) for 24 h at 37℃ under laser irradiation at certain time points. DOX was released quickly in the initial 8 h and reached a plateau region after 12 h. The release percentage of DOX in the pH 5.0 buffer was ~ 30%, significantly higher than that of 10% in the pH 7.4 buffer (Fig. 2B). And the laser irradiation (808 nm, 1.0 W/cm^2^, 5 min) obviously accelerated the DOX release. Around 60% of DOX was released in the pH 5.0 buffer after 24 h under the stimulation of laser irradiation, and the first irradiation at 2 h caused the mostly increased release of DOX, around 20% (Fig. 2B). Subsequently, we accessed the photothermal effect of TOADI in the physiological environment. We found that TOADI had a gradually increased temperature that stabilized at 49.4℃ after 5 min laser irradiation, whereas the temperature of free ICG reached 44.7℃ after 3 min irradiation and then slightly decreased to 43.5℃ at 5 min (Fig. 2C and **Figure S4**). It indicates that TOADI possesses a stronger photothermal effect and is more stable than free ICG. It was worth noting that the TOADI exhibited a higher temperature increase in comparison to the free ICG at the same concentration, which should be attributed to the increase of condensed concentration after ICG and DOX co-encapsulation. The PBS control and TOAD had no evident temperature increase after irradiation, meaning that TOAD did not generate the photothermal effect. Besides, the photostability of TOADI was tested. As shown in Fig. 2D, TOADI maintained higher photothermal performance and slower temperature decrease in each cycle compared with free ICG. Then, we evaluated the photothermal effect of different concentrations of TOADI from 10 to 20 µM. The quantification of infrared thermal images showed that the photothermal effect of TOADI was rising with the increase of its concentration (Fig. 2E&**F**). And with the increase of irradiation power (0.5, 1.0, and 1.5 W/cm^2^), the temperature of TOADI raised gradually (**Figure **S5). We confirm that TOADI has excellent properties for DOX loading and releasing, and performs stronger and more stable photothermal effect than free ICG.

### Codelivery of DOX and ICG by TOADI to tumor cells

**Fig. 3:**
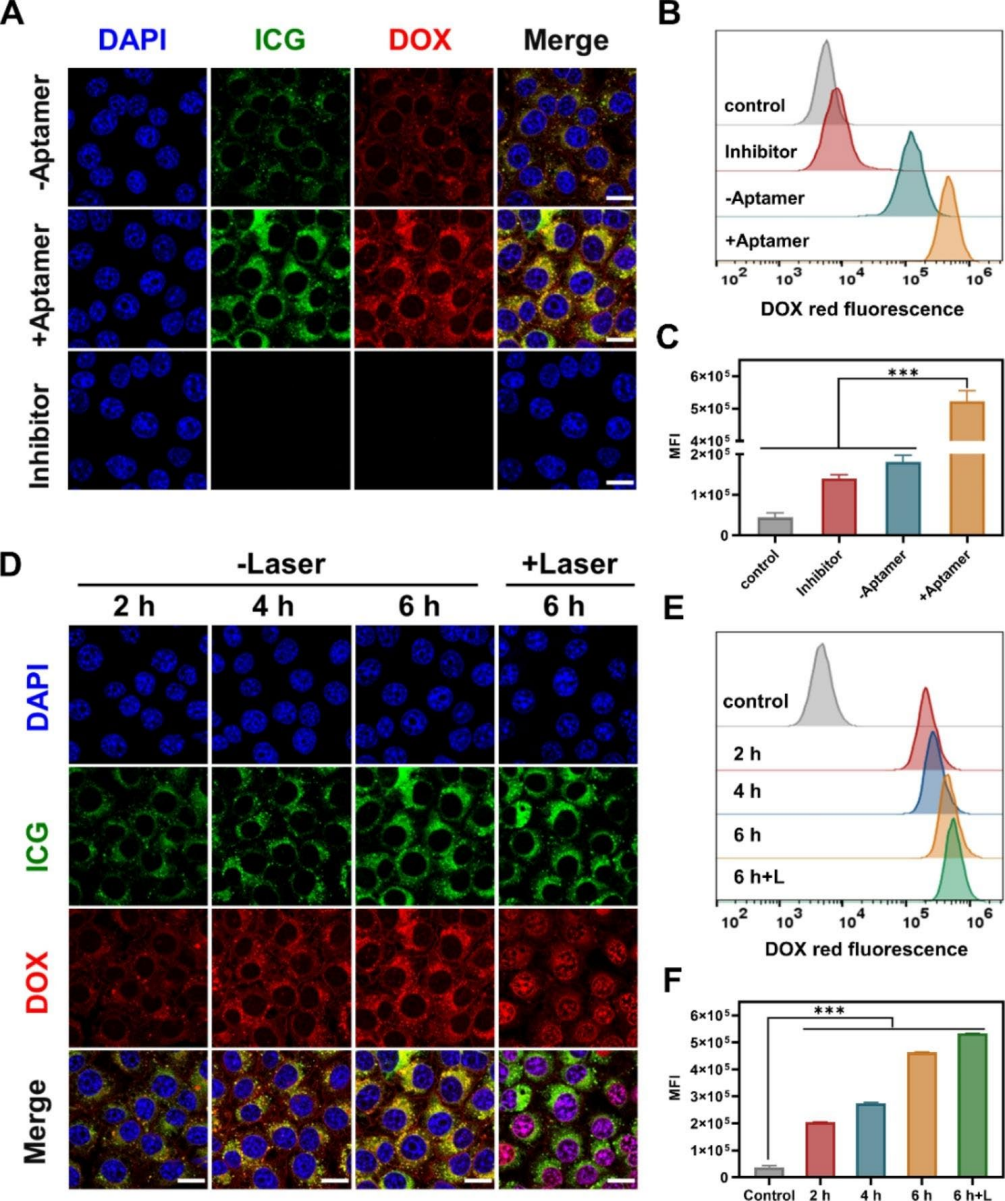
In vitro cellular uptake triggered by the DNA nanostructure-based drugs codelivery system. **(A)** Confocal images and **(B)** flow cytometry analysis of 4T1 cells incubated for 6 h with three different conditions: DNA origami nanovehicle without AS1411, nanovehicle with AS1411, and nanovehicle with AS1411 as well as endocytosis inhibitor. (**C**) Mean fluorescence intensity (MFI) of 4T1 cells from flow cytometry analysis in (**B**). (**D**) Confocal fluorescence images and (**E**) flow cytometry analysis of 4T1 cells after incubation with TOADI for 2, 4, and 6 h without laser irradiation and 6 h with laser irradiation (808 nm, 1.0 W/cm^2^, 5 min). (**F**) MFI of 4T1 cells from flow cytometry analysis in (**E**) (excitation wavelength: 480 nm for DOX, pseudocolor red; 780 nm for ICG, pseudocolor green; scale bar = 20 μm). The data are presented as the mean ± SD (n = 3). ***p < 0.001

Motivated by the outstanding properties of TOADI for drug release and photothermal effect, then we applied TOADI to the intracellular delivery of DOX and ICG into tumor cells for enhanced cancer therapy. First, we tested the stability of DNA origami in the physiological environment. DNA origami was incubated in cell culture medium with 10% fetal bovine serum (FBS) for 48 h, then detected by agarose gel electrophoresis. The results showed that about 84% of DNA origami remained stable after 24 h incubation in cell culture medium (**Figure **S6). DNA origami with hundreds of hybrid staples in a compact space can impede its degradation because of the intrinsic steric hindrance [29]. Therefore, DNA origami is stable enough as a drug nanocarrier for intracellular delivery. Subsequently, to explore the tumor-targeting ability of the nanosystem, 4T1 breast cancer cells were incubated with TOADI (+ AS1411 aptamers) and TODI (-AS1411 aptamers) respectively. 4T1 cells incubated with the TOADI (+ aptamer) exhibited obviously higher red (DOX) and green (ICG) fluorescence intensities than that incubated with TODI (-aptamer) after 6 h incubation (Fig. 3A). Further flow cytometry analysis showed that the mean fluorescence intensity (MFI) of TOADI in 4T1 cells was approximately 3-folds higher than that of TODI, meaning that the AS1411 aptamer improved at least threefold targeted cellular uptake of nanocarriers (Fig. 3B&**C**). Meanwhile, treatment with the endocytosis inhibitor blocked the internalization of TOADI causing almost no fluorescence in 4T1 cells. These data demonstrate that AS1411 aptamer can significantly reinforce the targeting ability of DNA origami and consequently enhance the internalization level in cancer cells.

To further explore the cellular uptake of TOADI in cancer cells, 4T1 cells were incubated with TOADI (corresponding to DNA origami: 0.4 nM, DOX: 20 µM, ICG: 14 µM) and detected at 2 h, 4 h, and 6 h. The confocal imaging results showed that the DOX and ICG fluorescence intensities in 4T1 cells were both gradually enhanced with the increase of incubation time (Fig. 3D), suggesting that more TOADI were internalized. The flow cytometry analysis of 4T1 cells showed the same result that the internalization level of TOADI increased after longer incubation (Fig. 3E&**F**). The laser irradiation (808 nm, 1.0 W/cm^2^) was executed at 6 h for 5 min. The fluorescence of DOX is mostly concentrated in the region of the nucleus after laser irradiation rather than in the cytoplasm (Fig. 3D). At the molecular level, the ingested TOADI by endocytosis degrades to release DOX that enter the nucleus [16]. Before laser irradiation, the release of DOX from the DNA origami relies on slow and passive degradation in an acidic endosomal environment. However, the aggregation of DOX in the nucleus induced by laser irradiation indicates that the photothermal effect of ICG significantly accelerates the release of DOX from TOADI in tumor cells. The photothermal effect of ICG triggered by laser irradiation not only is a switch to manually control the release of DOX but also functions as a photothermal therapy to induce the apoptosis of cancer cells. Therefore, the TOADI has great potential in manually-controlled chemo-phototherapy.

### Synergistic chemo-phototherapeutic efficacy in vitro

**Fig. 4:**
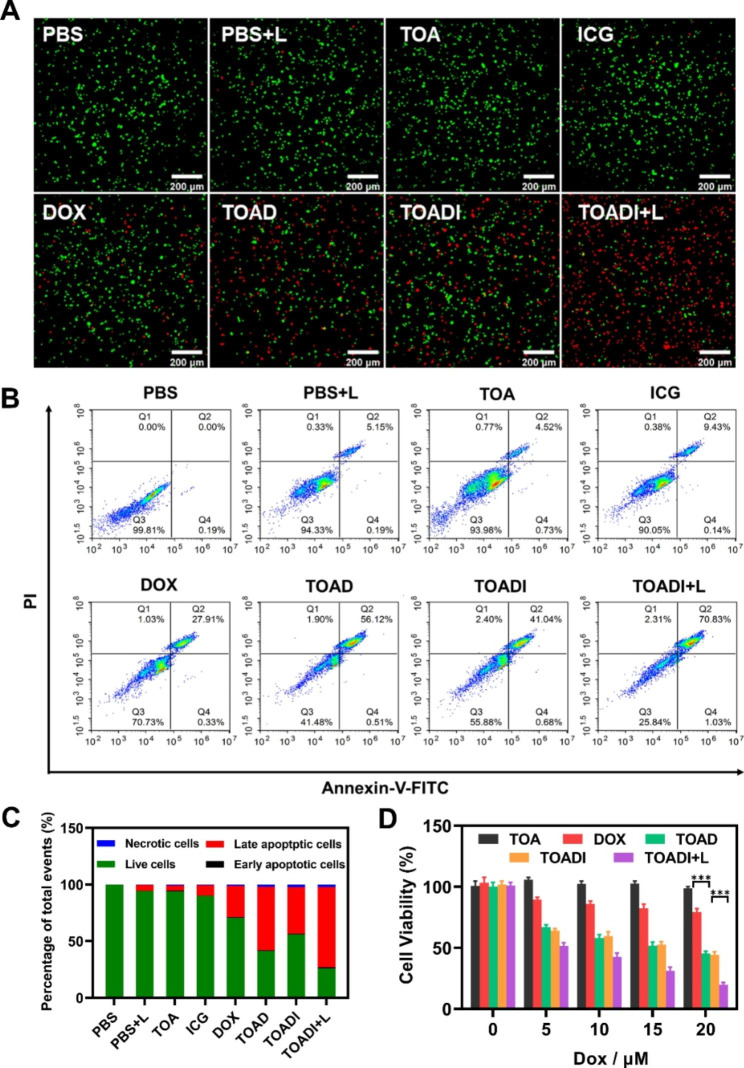
In vitro evaluation of synergistic PTT/chemotherapy in 4T1 cells. (**A**) Fluorescence images of calcein-AM/PI double-staining 4T1 cells for 24 h. (**B**) Flow cytometry analysis of cell apoptosis in 4T1 cells treated with different formulations for 24 h. (**C**) Comparisons of apoptosis and necrosis cell percentages. (**D**) Cell viability of 4T1 cells treated with TOA, DOX, TOAD, TOADI, and TOADI + L for 24 h, respectively (“+L” means with 808 nm laser irradiation). The data are presented as the mean ± SD (n = 3). ***p < 0.001

To study the synergistic chemo-phototherapy of TOADI in vitro, 4T1 cells were divided into 8 groups treated with different formulations respectively for 24 h. As shown in Fig. 4A, TOA and free ICG did not generate toxic effects; TOAD and TOADI caused more dead/late apoptotic cells than free DOX, suggesting that loading DOX on TOA enhanced the cellular uptake of DOX. The group treated by TOADI with laser irradiation (TOADI + L) had the most dead/late apoptotic cells, showing that the photothermal effect of ICG promoted the therapeutic effect of TOADI. To further quantify the therapeutic effect of these different formulations, the percentage of live and apoptotic cells for each group were labeled by staining of propidium iodide (PI) and Annexin V-FITC, then detected by flow cytometry (Fig. 4B). The quantification showed that the percentage of necrotic and apoptotic cells in the TOA group was almost negligible, indicating that the blank DNA origami with aptamers was not able to induce cell apoptosis. Notably, an increased proportion of apoptotic cells was detected in TOAD and TOADI groups, 57% and 42% respectively, compared with that 28% in the free DOX group. And the TOADI + L group had the highest rate of apoptotic cells (72%) (Fig. 4C). Also, we performed the cell counting kit-8 (CCK-8) assay to test the cell viability of drug-treated 4T1 cells. As shown in Fig. 4D, no matter how the concentration changed, the groups of TOADI and TOAD always had stronger cytotoxicity than the free DOX group, and the TOADI + L group had the lowest cell viability among all groups consistently. All the flow cytometry analysis and cell viability tests show two important points: (1) TOAD and TOADI both have stronger therapeutic effect than free DOX in the treatment of cancer, but there is no significant difference between them. (2) TOADI will perform a better therapeutic effect after laser irradiation. The findings indicate that the AS1411-modified functionalized DNA origami can efficiently codeliver DOX and ICG targeting tumor cells, which first enhances the chemotherapeutic effect of DOX; then the photothermal effect of ICG triggered by NIR laser irradiation activates the release of DOX from DNA origami, consistent with the conclusion of cellular uptake in vitro, which significantly improves the synergistic chemo-phototherapeutic efficacy.

### Apoptosis induced by TOADI

**Fig. 5:**
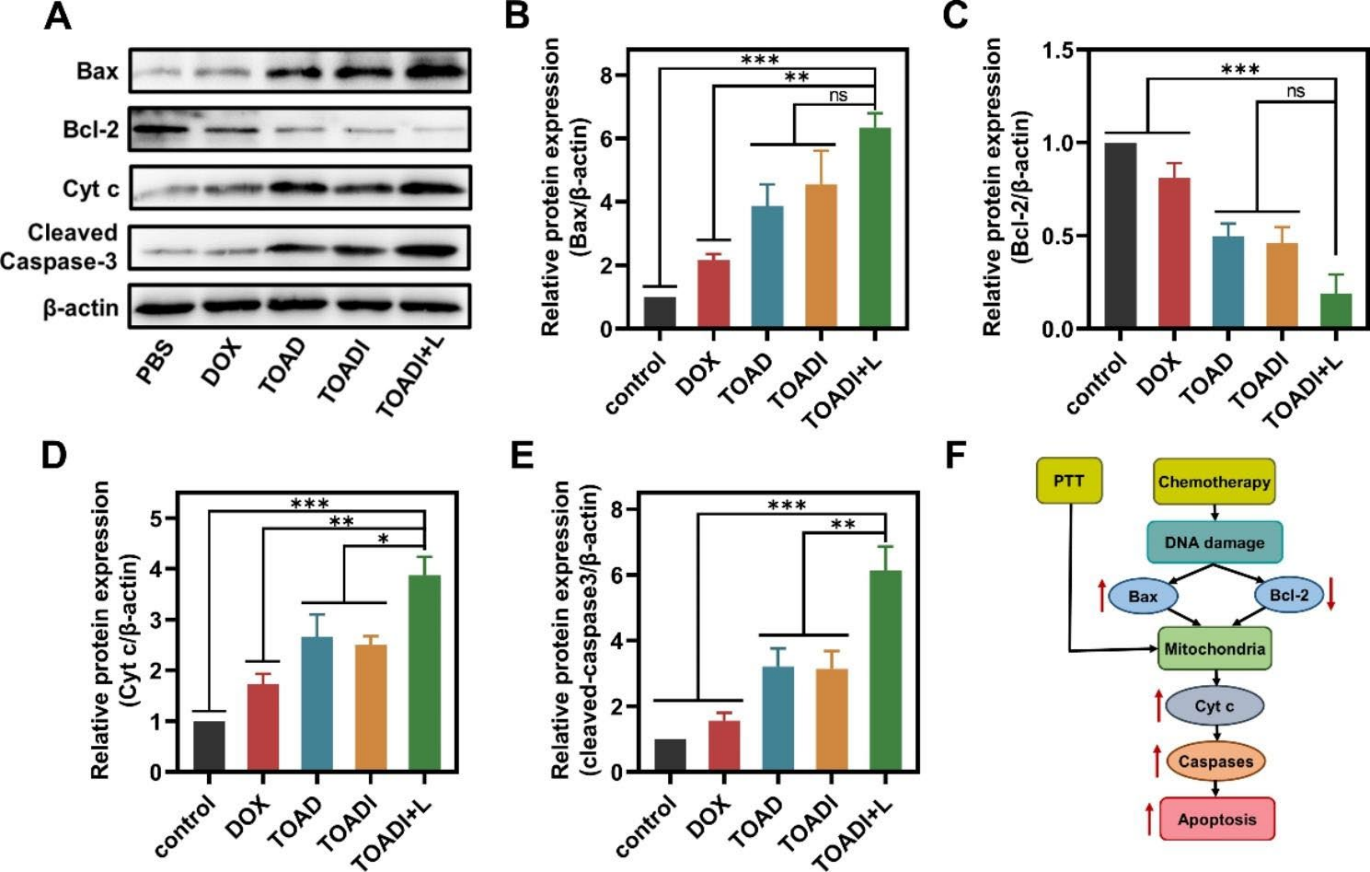
Intrinsic signal pathways analysis. (**A**) Western blot analysis of the protein expression of Bax, Bcl-2, Cyt c, and cleaved caspase-3 in 4T1 cells treated at different conditions. (**B**, **C**, **D** and **E**) The corresponding semi-quantitative analysis of Western blot band gray values of Bax, Bcl-2, Cyt c, and cleaved caspase-3 by ImageJ. (**F**) Schematic diagram of DNA origami nanovehicle inducing apoptosis via signaling pathways in vitro. The data are presented as the mean ± SD (n = 3). ns: not significant. *p < 0.05, **p < 0.01, ***p < 0.001

We further explored how TOADI affected the apoptotic pathway at the molecular level. B-cell lymphoma-2 (Bcl-2) and Bcl-2 associated X protein (Bax) are antiapoptotic and proapoptotic factors, respectively [44, 45]. During the intrinsic apoptosis, Bax induces the release of cytochrome c (Cyt c) from mitochondria, whereas Bcl-2 inhibits this progress [40, 46]. Cyt c works with other components to progress procaspase-3 into activated cleaved caspase-3 which plays an important role in subsequent cell apoptosis [47–49]. 4T1 cells were treated with free DOX, TOAD, TOADI, and TOADI + L, respectively. After 12 h, we harvested cells and performed the western blot analysis of the expression level of Bax, Bcl-2, Cyt c, and cleaved caspase-3. The expression of Bax, Cyt c, and cleaved caspase-3 was obviously increased and Bcl-2 was obviously decreased in the TOADI + L treated group (Fig. 5A). After the quantification of the expression of the four proteins, we found that the expression of Bax was significantly improved, while the expression of Bcl-2 was remarkably reduced after the treatment of TOADI + L compared with the groups of PBS control and DOX (Fig. 5B&**C**). For the expression of Bax and Bcl-2, although no statistical difference was found between the TOADI + L treatment and the TOAD/TOADI treatments in vitro, they showed a significant difference (*p* < 0.05) in 4T1 tumor-bearing mice (**Figure S14**). The expression of Cyt c and cleaved caspase-3 both exhibited a remarkable increase in the TOADI + L treated group compared with the other four groups (Fig. 5D&**E**). In summary, the synergistic chemo-phototherapeutic efficacy of TOADI induces the increased expression of Bax and decreased expression of Bcl-2 to promote the release of Cyt c from mitochondria which then activates more caspase-3 for cell apoptosis (Fig. 5F). On the other hand, the change of the expression level of apoptosis-related proteins also demonstrates that after the release of DOX from DNA origami triggered by laser irradiation, the TOADI exhibits a stronger synergistic chemo-phototherapeutic effect.

### Biodistribution and tumor imaging in vivo

**Fig. 6:**
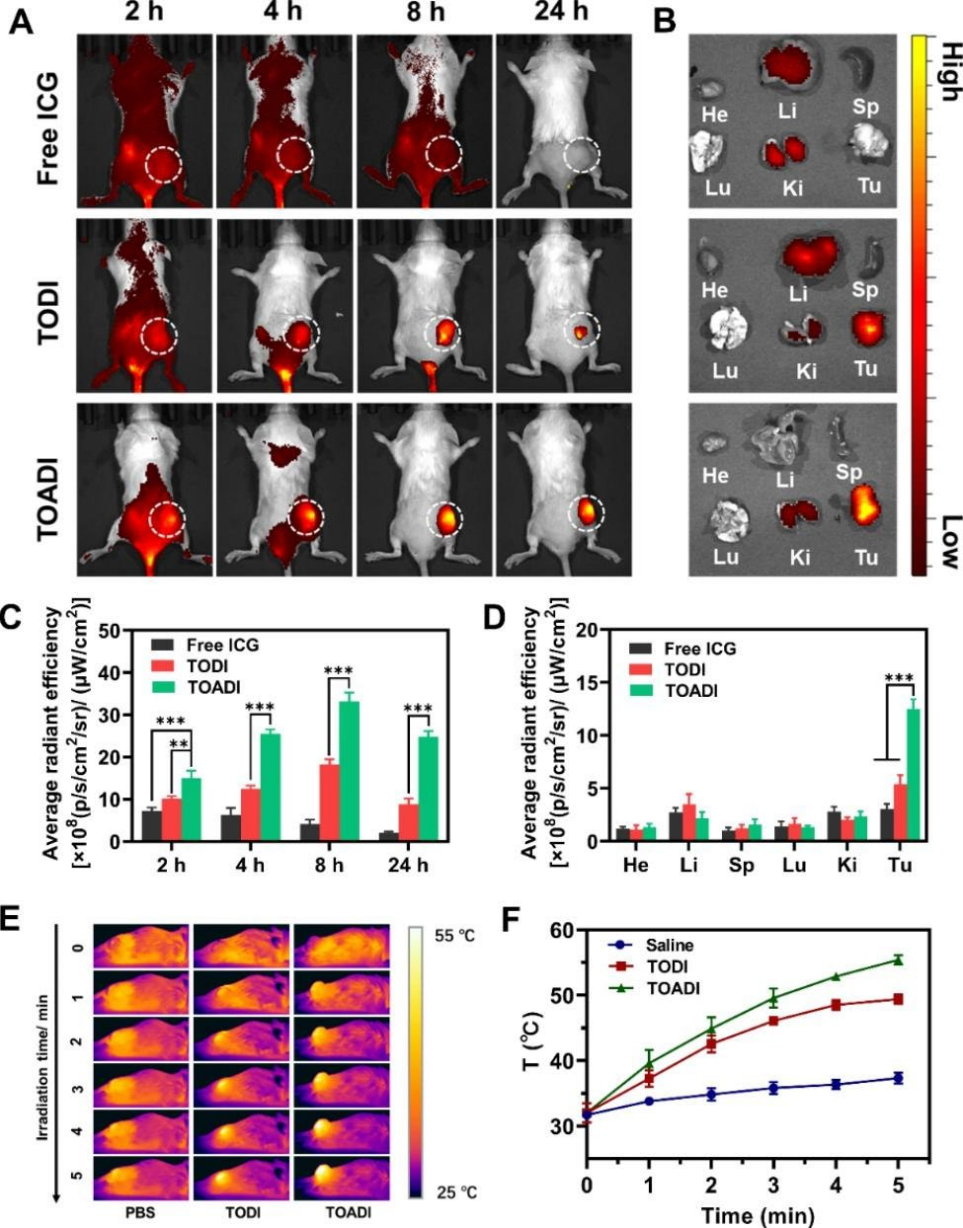
In vivo biodistribution and photothermal imaging. ICG was served as an imaging agent to create in vivo fluorescence imaging. (**A**) In vivo fluorescence images at 2, 4, 8, and 24 h and (**B**) Fluorescence photographs of ex vivo tissue and main organs of 4T1 tumor-bearing mice at 24 h post-injection after tail vein injection with free ICG, TODI, and TOADI. (Sp, Ki, He, Lu, Li, and Tu represent for spleen, kidney, heart, lung, liver, and tumor, respectively) (**C**) Semi-quantitative biodistribution of free ICG, TODI and TOADI in mice determined by the MFI of the tumor, respectively and semi-quantitative analysis (**D**) of tumors and major organs at 24 h post-injection. (**E**) Infrared thermal imaging and (**F**) temperature change curves of tumors from mice treated with PBS, TODI and TOADI under 808 nm laser irradiation. The data are presented as the mean ± SD (n = 3). **p < 0.01, ***p < 0.001

Besides photothermal therapy, ICG is also a superior indicator of fluorescence and photothermal imaging to study the biodistribution of TOADI in vivo. To evaluate its performance in intravital imaging, PBS, TODI, or TOADI was intravenously injected into 4T1 tumor-bearing mice. Then, the mice were scanned at various time points within 24 h to obtain real-time biodistribution fluorescence images, and at last, mice were euthanatized to harvest heart, liver, spleen, lung, kidney, and tumors for further ex vivo fluorescence imaging. As expected, no ICG fluorescence signals were detected at the tumor region in the free ICG treatment group after 24 h, and signals were observed in the liver and kidney predominantly compared with other organs, suggesting the fast elimination of free ICG from the body. In contrast, the ICG fluorescence signals were evidently observed in the tumor region in the TOADI treated group. Although the group of TODI was also observed stronger signals than the free ICG group in the tumor region, its fluorescence intensity was significantly weaker than that of the TOADI group at each time point (Fig. 6A&**C**). The analysis of the ICG fluorescence intensity of the organs revealed that the accumulation of TOADI in the tumor region was about four times more than that of free ICG and 2.5 times more than that of TODI (Fig. 6B&**D**). The higher accumulation of TODI than that of free ICG was attributed to the enhanced permeability and retention (EPR) effect of nanocarriers. These findings demonstrate that the loading of ICG on AS1411-guided functionalized DNA origami remarkably improves the actively targeted delivery of ICG into tumor cells, and presents a distinguishable signal at appropriate timing for next NIR imaging-guided PTT therapy. Subsequently, we detected photothermal imaging in 4T1 tumor-bearing mice. After laser irradiation, infrared thermal imaging and corresponding quantification of temperature changes in the tumor region showed that TOADI treatment caused the strongest photothermal effect which made the tumor region reach 55℃, in contrast to the 37℃ in the PBS treated group (Fig. 6E&**F**). These photothermal images also suggested that TOADI specifically targeted tumor cells and accumulated in the tumor region. Overall, TOADI exhibits outstanding superiority in targeting tumor cells in vivo for cancer therapy, as well as great value in fluorescence and photothermal imaging.

### Therapeutic effect of TOADI in vivo

**Fig. 7:**
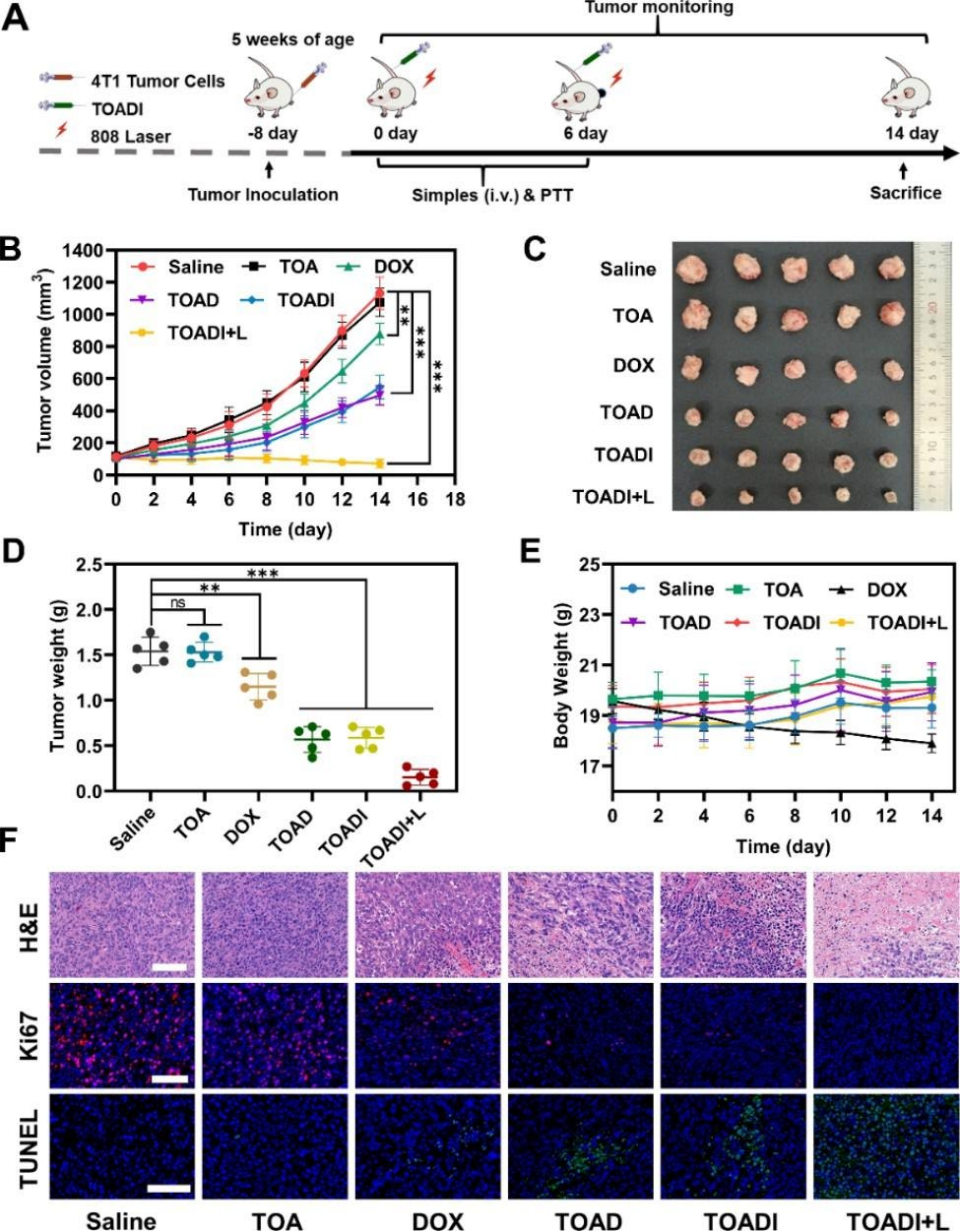
Therapeutic effects of the DNA origami-based nanovehicle in vivo. (**A**) Schematic timeline illustration of animal therapy. (**B**) Tumor volume of 4T1 tumor-bearing mice after the specified treatments. (**C**) Photographs of tumor tissues removed from groups treated with different formulations after 14 d. (**D**) Ex vivo tumor weights of each group weights at the end of the study (day 14). (**E**) Body weight curves of 4T1 tumor-bearing mice for each group. (**F**) Representative images of H&E, TUNEL, and Ki67 immunostaining of tumor tissues from different groups after various treatments. (scale bar = 100 μm). The data are presented as the mean ± SD (n = 5). **p < 0.01, ***p < 0.001

The synergistic chemo-phototherapy of TOADI was further evaluated in vivo. 4T1 tumor-bearing mice were randomly divided into six treatment groups: (i) Saline, (ii) TOA, (iii) DOX, (iv) TOAD, (v) TOADI, (vi) TOADI + L, and each group contains 5 mice. The mice were intravenously injected with different formulations and irradiated by laser as shown in Fig. 7A. We measured the tumor size and body weight every two days for total 14 days. As shown in Fig. 7B and **Figure S7**, tumors in the TOA treatment group grew as rapid as the saline control group, suggesting that TOA itself had no significant effect on tumor growth. Additionally, a little stronger tumor inhibition effect of TOAD and TOADI than the free DOX was due to the targeted delivery of DOX to tumor cells by TOA. Although free DOX, TOAD, and TOADI exhibited slight suppression of tumor growth compared with the saline control, tumors kept exacerbating. By contrast, the tumor volume gradually became smaller after TOADI + L treatment (Fig. 7B), demonstrating the outstanding therapeutic effect of TOADI + L against tumors. After 14 days, tumors excised from mice bodies directly revealed that the TOADI + L treated group had the smallest tumors than other groups (Fig. 7C&**D**), showing approximately 90% of tumor inhibition. Meanwhile, except for the free DOX group, other groups had no significant decrease in body weight during experiments (Fig. 7E), meaning that the targeted delivery of DOX was able to reduce its cytotoxic effects on normal cells [50].

Then, we performed the H&E, TUNEL, and Ki67 immunostaining of tumor slices to further analyze the antitumor effects and biosafety. In the H&E staining, it was observed that the TOADI + L treatment group caused more necrotic regions compared with the normal nuclear structure in the control group (Fig. 7F and **Figure S8**). And TOAD and TOADI treatment caused moderate tumor tissue damage. Additionally, the TUNEL and Ki67 immunostaining reflected the apoptosis and proliferation of tumor cells respectively. The TOADI + L treated group had the highest amount of (TUNEL) green signals compared with the other groups, meaning that TOADI + L treatment caused most cell apoptosis. Moderate apoptotic signals appeared in the TOAD and TOADI groups, whereas just a few apoptotic tumor cells were found in the free DOX treatment group (Fig. 7F and **Figure S9**). Similarly, TOADI + L treated group had the lowest amount of (Ki67) purple signals, demonstrating the remarkably reduced proliferation of tumor cells (Fig. 7F and **Figure S10**). All these results indicate that the synergistic chemo-phototherapy of TOADI has enormous potential to be applied for efficient cancer treatment.

The biosafety of TOADI in vivo was subsequently evaluated. H&E staining of major organs showed that TOADI + L treatment did not cause obvious histological damage, suggesting that it was biosafe for normal tissues (**Figure **S11). Blood samples from representative groups (PBS, TOADI and TOADI + L) were collected for the blood biochemistry and hematology examination. Markers of liver and kidney function had no significant difference between the TOADI + L group and the control, indicating that the TOADI + L treatment did not cause hepatic or kidney disorders (**Figure **S12). Furthermore, the serum indexes maintained in the normal range after TOADI + L treatment, indicating its inappreciable toxicity (**Figure **S13). Thus, the low systemic toxicity of TOADI makes it promising for future biomedical applications in the clinic.

In addition, we investigated the expression of Bax, Bcl-2, Cyt c, and cleaved caspase-3 in tumor sections of treated mice at day 14. As shown in **Figure** S14A and **Figure** S14B, the TOADI + L treated group had significantly higher expression of Bax, Cyt c, and cleaved caspase-3, and lower expression of Bcl-2 than other groups. The change of the expression of the four apoptosis-related proteins was consistent with the results of the western blot analysis of 4T1 cells in vitro. These results indicate that TOADI can be successfully transported into tumor cells, and efficiently function against tumors after laser irradiation.

## Conclusions

In summary, we have successfully developed a multifunctional nanovehicle called TOADI for targeted codelivery of DOX and ICG into cancer cells, which can be applied for synergistic chemo-phototherapy of cancers. Our results show that the DNA origami with AS1411 aptamers is able to efficiently codeliver DOX and ICG to tumor sites. In vitro experiments show that the release of DOX from TOADI is triggered by NIR irradiation, and the acidic environment of lysosomes/endosomes facilitates the release. The in vitro and in vivo anticancer assays all verify that TOADI can significantly increase its accumulation in tumor region and enhance cancer therapeutic effects for breast cancer. Moreover, to our knowledge, this is the first report of co-loading DOX and ICG on DNA origami. With these excellent advantages, this biocompatible and multifunctional DNA origami-based nanovehicle has enormous potential for loading other oligonucleotides, imaging agents, and chemotherapeutic drugs for cancer therapy and diagnosis.

## Electronic supplementary material

Below is the link to the electronic supplementary material.

Supplementary Material 1

## Data Availability

All data generated or analyzed during this study are included in this current article and its additional files.
