# Supplementary material for: NIR/pH-triggered aptamer-functionalized DNA origami nanovehicle for imaging-guided chemo-phototherapy
Source: J Nanobiotechnology. 2023 Jun 10;21:186. doi: 10.1186/s12951-023-01953-9 (PMC10257293; doi:10.1186/s12951-023-01953-9)
Supplement: Supplementary file 1 — Supplementary Material 1 [file 12951_2023_1953_MOESM1_ESM.docx]

**Supporting Information**

**NIR/pH-triggered Aptamer-functionalized DNA Origami Nanovehicle for Imaging-guided Chemo-phototherapy**

Mengyue Li ^1^, Geng Yang ^1^, Yue Zheng ^1^, Jiazhen Lv ^1^, Wanyi Zhou ^1^, Hanxi Zhang ^1^, Fengming You ^2^, Chunhui Wu ^1^, Hong Yang ^1 *^, Yiyao Liu ^1, 2 *^

*^1^ Department of Pharmacy, Personalized Drug Therapy Key Laboratory of Sichuan Province, Sichuan Provincial People’s Hospital, and School of Life Science and Technology, University of Electronic Science and Technology of China, Chengdu, P.R. China.*

*^2^ TCM Regulating Metabolic Diseases Key Laboratory of Sichuan Province, Hospital of Chengdu University of Traditional Chinese Medicine, No. 39 Shi-er-qiao Road, Chengdu 610072, Sichuan, P.R. China.*

**^*^Corresponding authors**

E-mail address: [yanghongyh@uestc.edu.cn](mailto:yanghongyh@uestc.edu.cn) (H. Yang), [liuyiyao@uestc.edu.cn](mailto:liuyiyao@uestc.edu.cn) (Y. Liu)

**Methods**

***1. DOX loading to DNA origami***

Briefly, DOX (2 mM) was incubated with triangular DNA origami with AS1411 (TOA, 20 nM) for 6, 12, 24 and 48 h under gentle shaking at room temperature to construct triangular DNA origami with AS1411 loading DOX (TOAD). After loading, the red mixture was centrifuged at 10000 rpm at room temperature for 10 min and washed three times to remove the unloaded DOX. And the free DOX in the supernatant was quantified by measuring the absorption at 480 nm. Lastly, the loading efficiency of DNA origami to DOX was calculated by the following equation: Loading efficiency of DOX into TOA = [(DOX added - DOX unloaded) / DOX added] × 100%. After 12 h incubation, loading efficiency can reach 50%. DOX-loaded triangular DNA origami with AS1411 (TOAD) was then redissolved in PBS to form a stock solution (1 mM DOX and 20 nM triangular DNA origami with AS1411).

***2. ICG loading on DNA origami***

ICG solutions (1mM in ddH_2_O) were incubated with triangular DNA origami with AS1411 loading DOX (TOAD, 20 nM triangular DNA origami and 1mM DOX in PBS) for 2, 6, 12 and 48 h under gentle shaking at room temperature to construct triangular DNA origami with AS1411 loading DOX and ICG (TOADI) in dark condition. After loading, the green mixture was centrifuged at 10000 rpm at room temperature for 10 min and washed three times to remove the unloaded ICG. And the free ICG in the supernatant was quantified by measuring the absorption at 780 nm. Lastly, the loading efficiency of DNA origami to ICG was calculated by the following equation: Loading efficiency of ICG into TOAD = [(ICG added - ICG unloaded) / ICG added] × 100%. After 6 h incubation, loading efficiency can reach 70%. DOX and ICG-loaded triangular DNA origami with AS1411 (TOADI) was then redissolved in PBS to form a stock solution (1 mM DOX, 700 μM ICG, and 20 nM triangular DNA origami with AS1411).

***3. DOX release efficiency***

To explore the DOX release behavior, DOX and ICG-loaded triangular DNA origamis with AS1411 (TOADI) were incubated in PBS at pH 7.4 (approximate the blood pH value) or 5.0 (approximate the acidic endosomes/lysosomes pH value) with or without laser (1.0 W/cm^2^, 5 min), respectively for 0, 2, 4, 6, 12 and 24 h at 37℃. The cumulative released DOX was quantified by measuring the absorption at 480 nm. The DOX release efficiency was calculated by the following equation: DOX release efficiency = (DOX cumulative released / DOX-loaded) ×100%.

***4. Photothermal effect of TOADI***

TOADI aqueous solution (1 mL) of different concentrations (corresponding to ICG: 10 μM, 15 μM and 20 μM) was irradiated by a NIR laser (808 nm, 1.0 W/cm^2^) for 5 min, and the thermal infrared (IR) imaging camera was used to monitor the temperature change of TOADI solutions. The photothermal effect of TOADI solution (corresponding to ICG concentration: 14 μM) under different powers of 808 nm NIR laser (0.5, 1.0 and 1.5 W/cm^2^) was determined by the same method. Meanwhile, the concentration of free ICG (14 μM) is the same as that of TOADI. The PBS, ICG and TOAD aqueous solutions were used as control samples. the temperature of the solution was also recorded.

***5. Serum stability***

Triangular DNA origami was incubated in the 10% FBS solution (diluted with 1× PBS) for 0, 12, 24, 36 and 48 h at 37℃, respectively. Finally, the DNA residual was quantified by Image J analysis.

***6. Cell culture***

Mouse breast cancer cell line (4T1) cells were purchased from American Type Culture Collection (ATCC, Manassas, VA, USA). The cells were cultured in RPMI 1640 medium containing 10% FBS, penicillin (100 U/mL), and streptomycin (100 U/mL) in an atmosphere of 5% CO_2_ at 37℃.

***7. Intracellular uptake***

To observe the *in vitro* intracellular uptake behavior of triangular DNA origami with AS1411 loading DOX and ICG (TOADI), Briefly, 4T1 cells (1×10^5^ cells/well) were seeded in 35-mm confocal dishes and cultured overnight to enable cell-attached completely. And then the cells were subjected to a fresh culture medium comprising TOADI with a final triangular DNA origami concentration of 0.4 nM (corresponding to DOX: 20 μM, ICG: 14 μM) in 1mL RPMI 1640 complete culture medium for 2, 4 and 6 h without laser irradiation and 6 h with laser irradiation (808 nm, 1.0 W/cm^2^, 5 min). To further examine the *in vitro* specific recognition of AS1411 aptamer in DNA nanocarriers and nucleolin in the 4T1 cells. Briefly, 4T1 cells (1×10^5^ cells/well) were seeded in 35-mm confocal dishes and cultured overnight to enable cell-attached completely. Then, 4T1 cells incubated for 6 h with three different conditions: DNA origami nanovehicle without AS1411, nanovehicle with AS1411, and nanovehicle with AS1411 as well as endocytosis inhibitor. Dynasore was added into dishes 30 mins before the addition of nanovehicle with AS1411 to inhibit the endocytosis in advance.

After drug incubation, all cells were rinsed three times with PBS and fixed with 4% paraformaldehyde for 20 min. The nuclei were stained by 4′,6-diamidino-2-phenylindole (DAPI) according to protocols provided by manufacturers, and the fluorescence images were subsequently observed through a laser confocal fluorescent microscopy (Zeiss) with 480 nm excitation for DOX and 780 nm excitation for ICG in TOADI. 4T1 cells were collected to further quantify the endocytosis of TOADI in 4T1 cells after co-incubation via fluorescence-activated cell sorting (FACS) analysis.

***8. Cytotoxicity measurements***

The antitumor effect *in vitro* was studied by evaluating the viability of the cancer cells (4T1) using a CCK-8 assay. After seeding in 96-well plates (1×10^4^ cells per well) and culturing overnight, 4T1 cells were incubated with PBS, TOA, DOX, TOAD, TOADI, and TOADI+L in 100 μL RPMI 1640 complete culture medium for 24 h. Then the old medium was removed and all the groups were treated with the CCK-8 assay following the manufacturer's protocol. In the TOADI+L group, "+L" means cells were treated with 808 nm laser irradiation (1.0 W/cm^2^, 5 min) at 6 h. For each group, we set up 4 different final concentrations of triangular DNA origami (0, 0.1, 0.2, 0.3, or 0.4 nM, corresponding to DOX: 0, 5, 10, 15, or 20 μM, ICG: 0, 3.5, 7.0, 10.5, or 14 μM).

Except for the CCK-8 assay, live/dead cell staining assay was also used to evaluate cytotoxicity via CLSM. After seeding in a six-well plate (6×10^5^ cells per well) and culturing overnight. Subsequently, 4T1 cells were incubated with PBS, PBS+L, TOA, ICG, DOX, TOAD, TOADI, and TOADI+L at a final triangular DNA origami concentration of 0.4 nM (corresponding to DOX: 20 μM, ICG: 14 μM) in 1 mL RPMI 1640 complete culture medium for 24 h. After incubation, the cells were trypsinized for 3 min to obtain the cell suspension, and calcein-AM (1 μL)/propidium iodide (PI) (3 μL) was added to the cell suspension and incubated for 30 min. Finally, the cells were washed with PBS (pH 7.4) three times and imaged by CLSM. In the TOADI+L group, "+L" means cells were treated with 808 nm laser irradiation (1.0 W/cm^2^, 5 min) at 6 h.

The cytotoxic effect of the various treatments on 4T1 cells was also assessed by cell apoptosis analysis. Briefly, 4T1 cells were treated with PBS, PBS+L, TOA, ICG, DOX, TOAD, TOADI, and TOADI+L at a final triangular DNA origami concentration of 0.4 nM (corresponding to DOX: 20 μM, ICG: 14 μM) in 1 mL RPMI 1640 complete culture medium. After incubation was performed for another 24 h, the cells were trypsinized for 3 min to obtain the cell suspension and washed with PBS three times, then resuspended in 100 μL of binding buffer containing Annexin V-FITC (5 μL) and PI (5 μL) following the manufacturer’s instructions. The mixture was then incubated in the dark for 15 min and a further 400 μL of binding buffer was applied to it before the study was carried out. Finally, the DOX fluorescent was detected by the BD FACS Calibur flow cytometry system (BD Bioscience) with 480 nm excitation. In the TOADI+L group, "+L" means cells were treated with 808 nm laser irradiation (1.0 W/cm^2^, 5 min) at 6 h.

***9. Histological analysis*** ***and immunofluorescence***

To further verify the therapeutic effect and mechanism of nanodevice, the tumor tissue was collected, fixed with 4% paraformaldehyde for 24 h, embedded in paraffin, and then stained with hematoxylin and eosin (H&E), Ki67 kits (Abclonal, China) and TUNEL kits (Roche, China) to perform histological analysis of tumors. Sections were also immunostained with anti-Bax antibody (Bioss, China), anti-Bcl-2 antibody (Bioss, China), anti-Cyt c antibody (Abclonal, China), and cleaved caspase-3 antibody (Cell signaling technology, China) to investigate the expression level of the mitochondria apoptotic pathway-associated protein.

***10. Evaluation of the biocompatibility***

To evaluate the biocompatibility of the nanodevice, After the treatment, the mice were sacrificed and the major organs (heart, lung, spleen, liver, and kidney) were collected for H&E staining to evaluate the biocompatibility. Meanwhile, blood samples were also withdrawn from the mice for blood chemistry tests and routine blood analysis.

**Supplementary Figures**

**
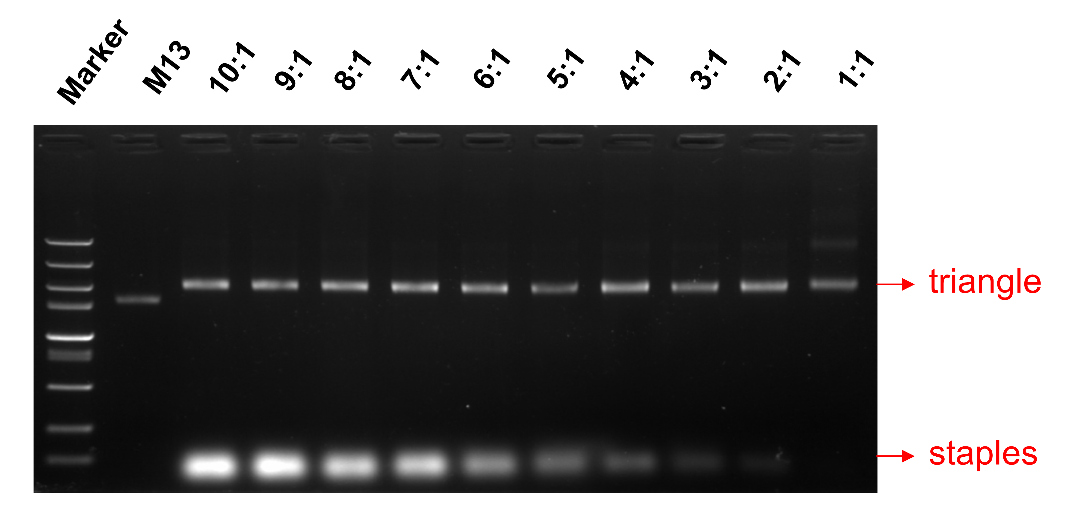
**

**Figure S1**. Optimization of the assembly conditions of triangle DNA origami. 1% agarose gel electrophoresis characterization of triangle DNA origami. Lane 1: Marker; Lane 2: M13mp18; Lane 3-12: The ratio of M13mp18 to staples for assembly of TO was as 10:1; 9:1; 8:1; 7:1; 6:1; 5:1; 4:1; 3:1; 2:1; 1:1, respectively.


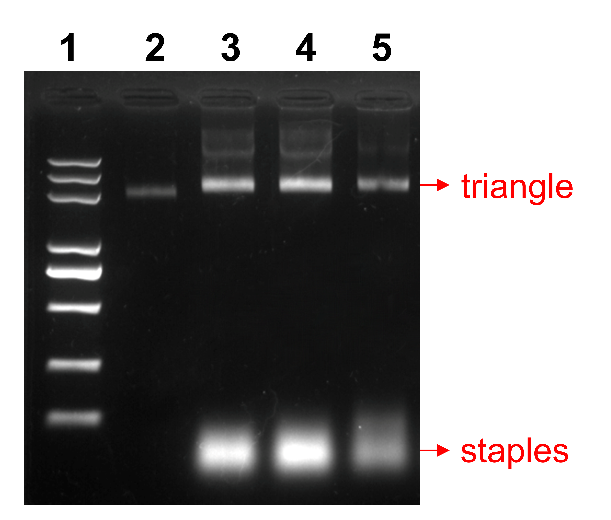


**Figure S2**. Optimization of the assembly conditions of triangle DNA origami modification with AS1411. 1% agarose gel electrophoresis characterization of the triangle DNA origami after modification with AS1411. Lane 1: Marker; Lane 2: M13mp18; Lane 3-5: The ratio of AS1411 to TO for assembly of TOA was as 1:6:24; 1:6:36; 1:6:12, respectively.


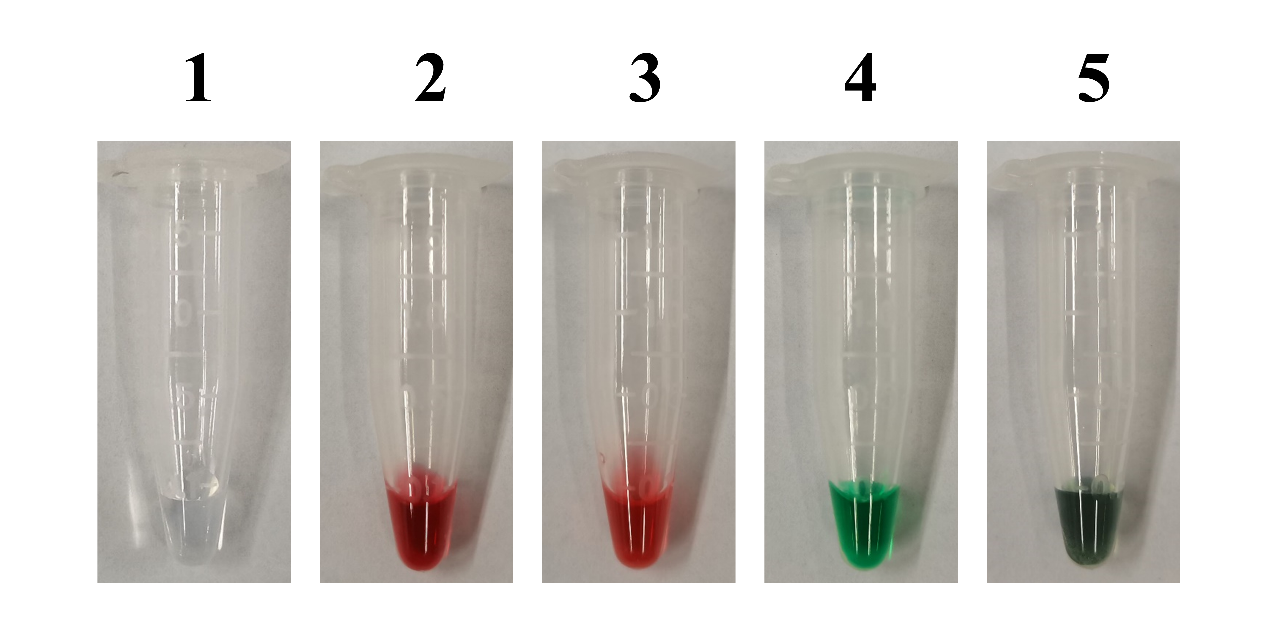


**Figure S3**. The photograph shows solutions of TOA, DOX, TOAD, ICG, and TOADI. (1: Triangle DNA origami with AS1411 (TOA); 2: DOX; 3: Triangle DNA origami with DOX (TOAD); 4: ICG; 5: Triangle DNA origami with DOX and the ICG(TOADI)).


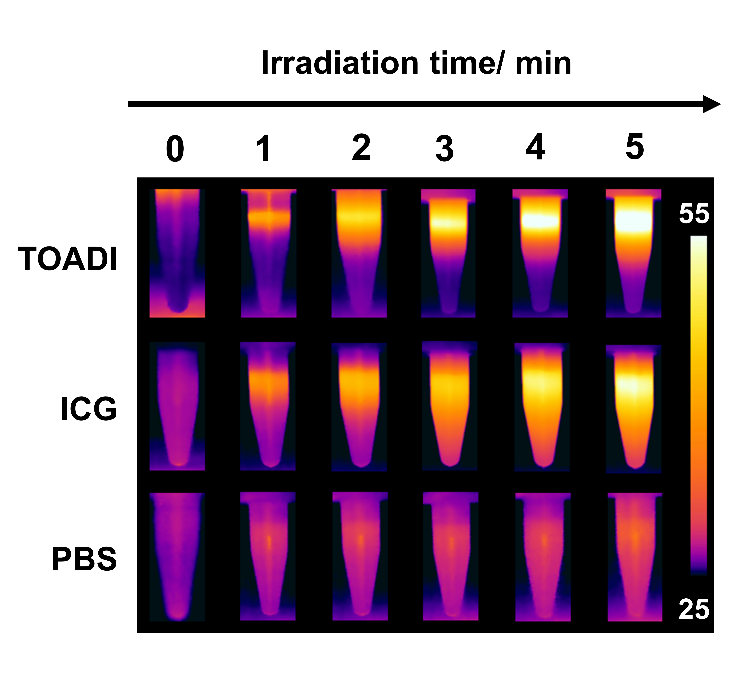


**Figure S4**. Infrared thermal images of TOADI, ICG, and PBS were used as control samples, and laser irradiation (808 nm, 1.0 W/cm^2^) was monitored by an infrared thermal camera *in vitro*.

**Figure S5**. Temperature curves of TOADI dissolved in PBS (corresponding to ICG:14 μM) under 808 nm laser irradiation with various power (0.5, 1.0, and 1.5 W/cm^2^). (TOADI: Triangle DNA origami with DOX and the ICG).


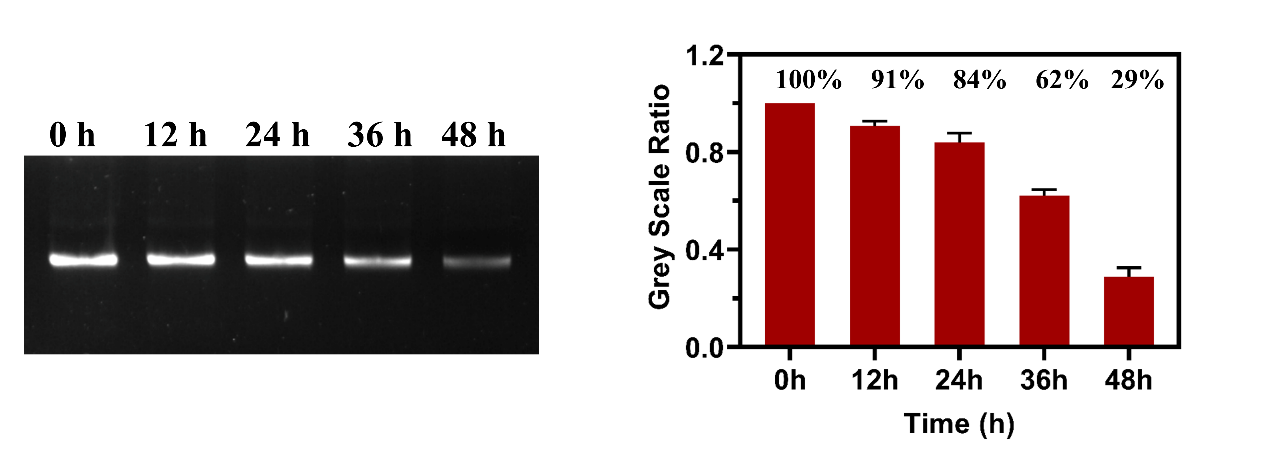


**Figure S6**. Stability of DNA origami. 1% agarose gel electrophoresis results showed that in the physiological environment, DNA origami bands were still clearly visible after 48 h of incubation. According to gray statistics, about 29% of DNA origami could maintain structural integrity after 48 h, which indicated that DNA origami could remain stable in the physiological environment for a long time without degradation.


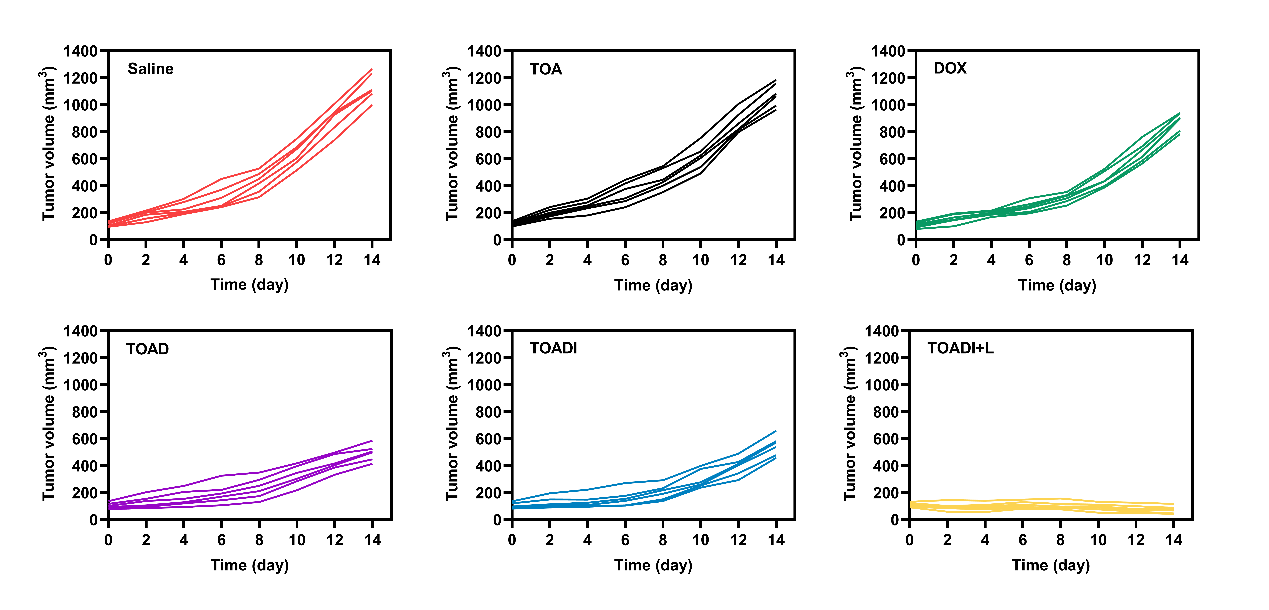


**Figure S7**. Individual tumor growth kinetics in different groups during various treatments.

**Figure S8**. Semi-quantification of tumor necrosis area. The data were shown as mean ± SD, n = 3 per group, ns: not significant, ***p* < 0.01, ****p* < 0.001.

**Figure S9**. Semi-quantification of TUNEL positive nucleus area. The data were shown as mean ± SD, n = 3 per group, ns: not significant, ****p* < 0.001.

**Figure S10**. Semi-quantification of KI67 positive nucleus area. The data were shown as mean ± SD, n = 3 per group, ns: not significant, ***p* < 0.01, ****p* < 0.001.


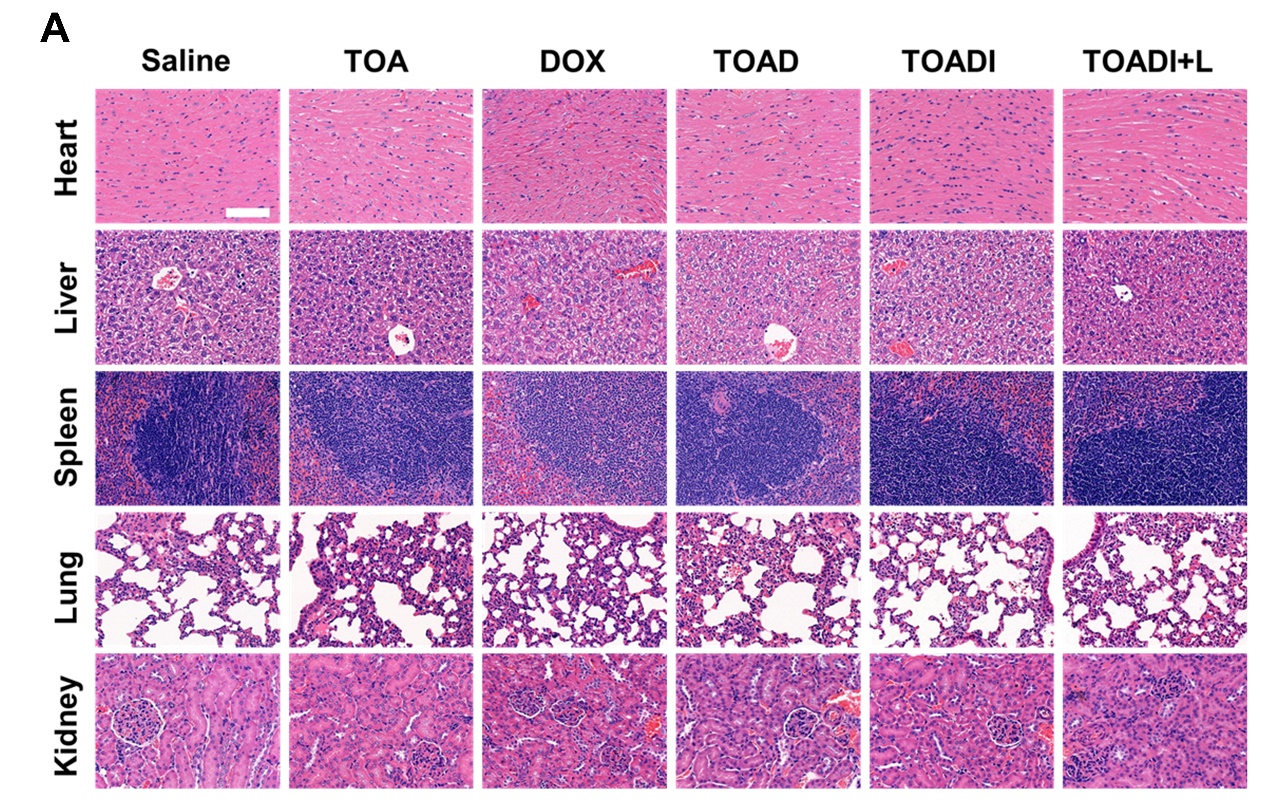


**Figure S11**. H&E-stained histological images of major organs (heart, liver, spleen, lung, and kidney) in tumor-bearing mice were collected at the end of different treatment groups (scale bar = 100 μm).


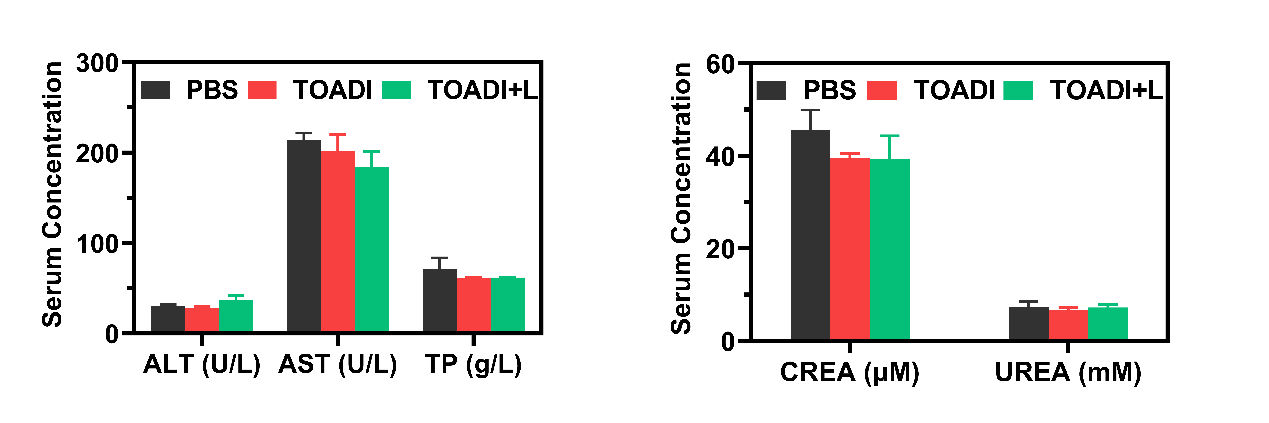


**Figure S12**. Biochemical parameters of mice at the end of treatment post i.v. injection of PBS and different groups of nanoparticles. The data are presented as the mean ± SD (n = 3).


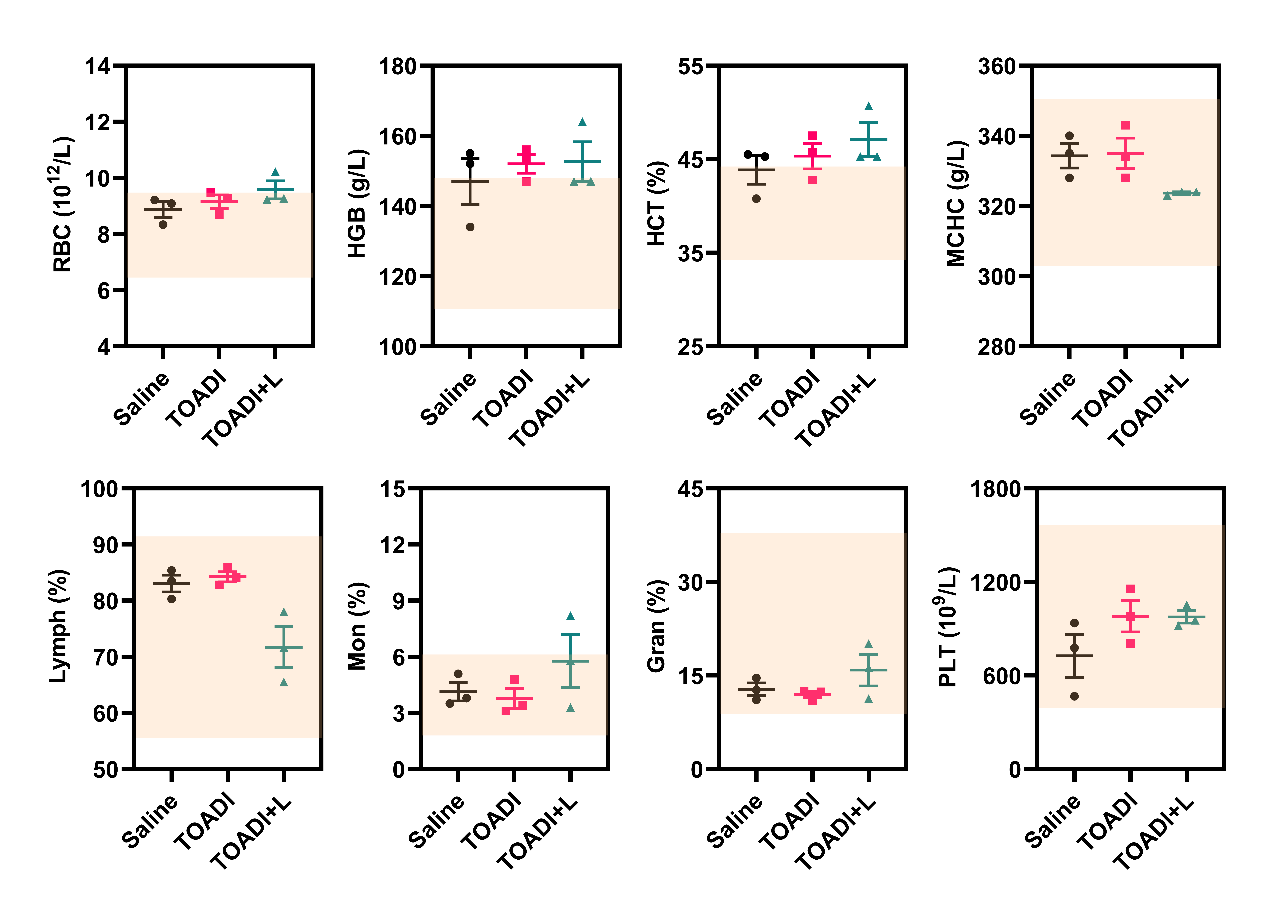


**Figure S13**. Complete blood count results (red blood cell (RBC), hemoglobin (HGB), hematocrit (HCT), mean corpuscular hemoglobin concentration (MCHC), Lymph, monocytes (Mon), Granulocytes (Gran), and platelet (PLT)) of mice after different treatments at day 14.


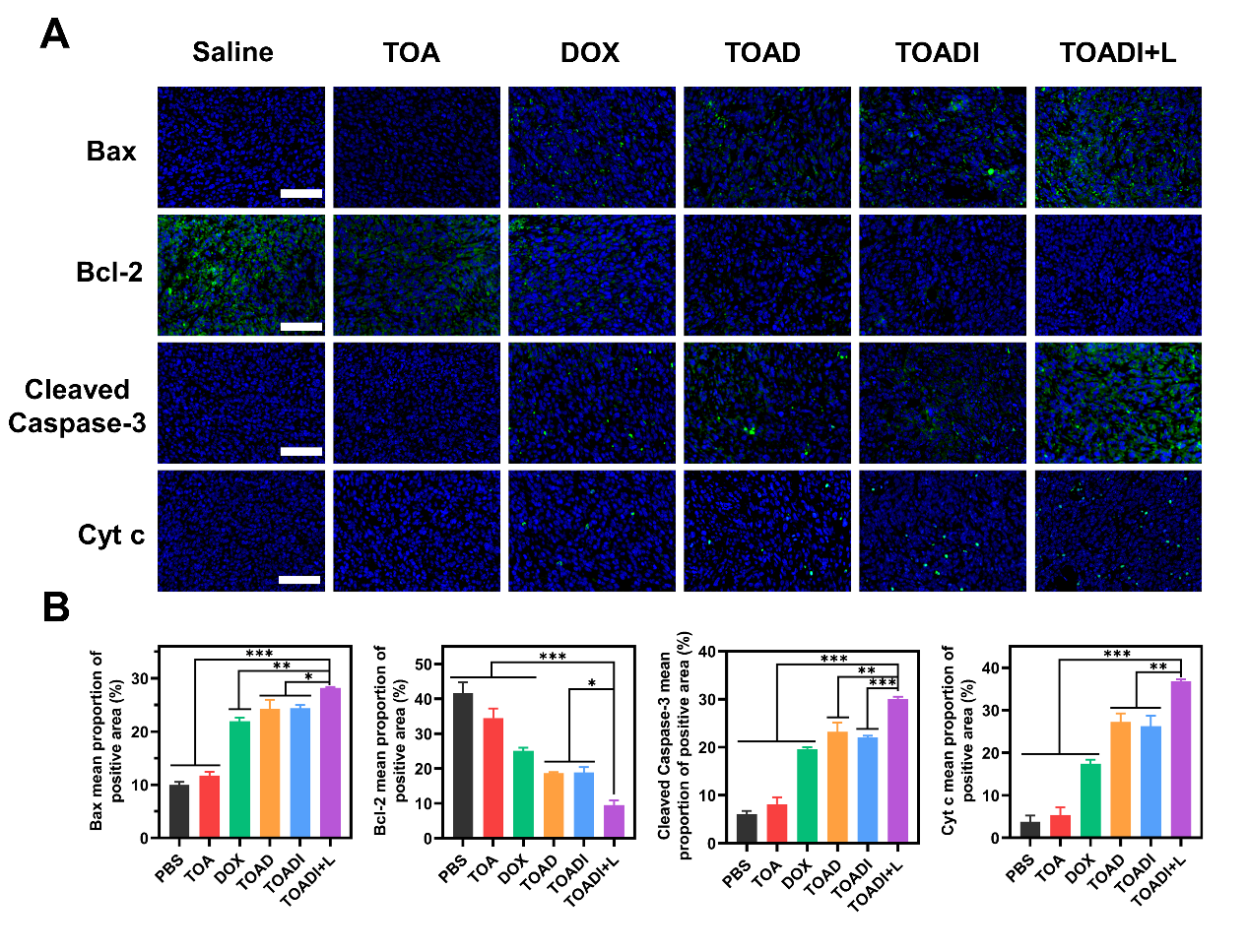


**Figure** **S14**. (**A**) Representative images of Bax, Bcl-2, Cleaved Caspase-3, and Cyt c immunostaining of 4T1 tumor-bearing mice from the different treatments, following 14 days of treatment (scale bar = 100 μm; Original magnification 20×). (**B**) The mean proportion of the positive area of Bax, Cleaved Caspase-3 and Bcl-2 of 4T1 tumor-bearing mice at the end of different treatment groups. The data was shown as mean ± SD (n = 3 per group), **p* < 0.05, ***p* < 0.01, ****p* < 0.001.

**Table S1**. Loading Efficiency of DOX into TOA.

| Time  (h) | TOA concentration  (nM) | Initial DOX concentration  (μM) | Loading DOX concentration  (μM) | Loading efficiency  of DOX into TOA  (%) |
| --- | --- | --- | --- | --- |
| 6 | 20 | 2000 | 612 | 30.6 |
| 12 | 20 | 2000 | 1018 | 50.9 |
| 24 | 20 | 2000 | 1270 | 63.5 |
| 48 | 20 | 2000 | 1576 | 78.8 |

**Table S2**. Loading Efficiency of ICG into TOAD

| Time  (h) | TOA concentration  (nM) | Initial ICG concentration  (μM) | Loading ICG concentration  (μM) | Loading efficiency  of ICG into TOAD  (%) |
| --- | --- | --- | --- | --- |
| 2 | 20 | 1000 | 609 | 60.9 |
| 6 | 20 | 1000 | 700 | 70 |
| 12 | 20 | 1000 | 717 | 71.7 |
| 48 | 20 | 1000 | 736 | 73.6 |

**4. DNA sequences**


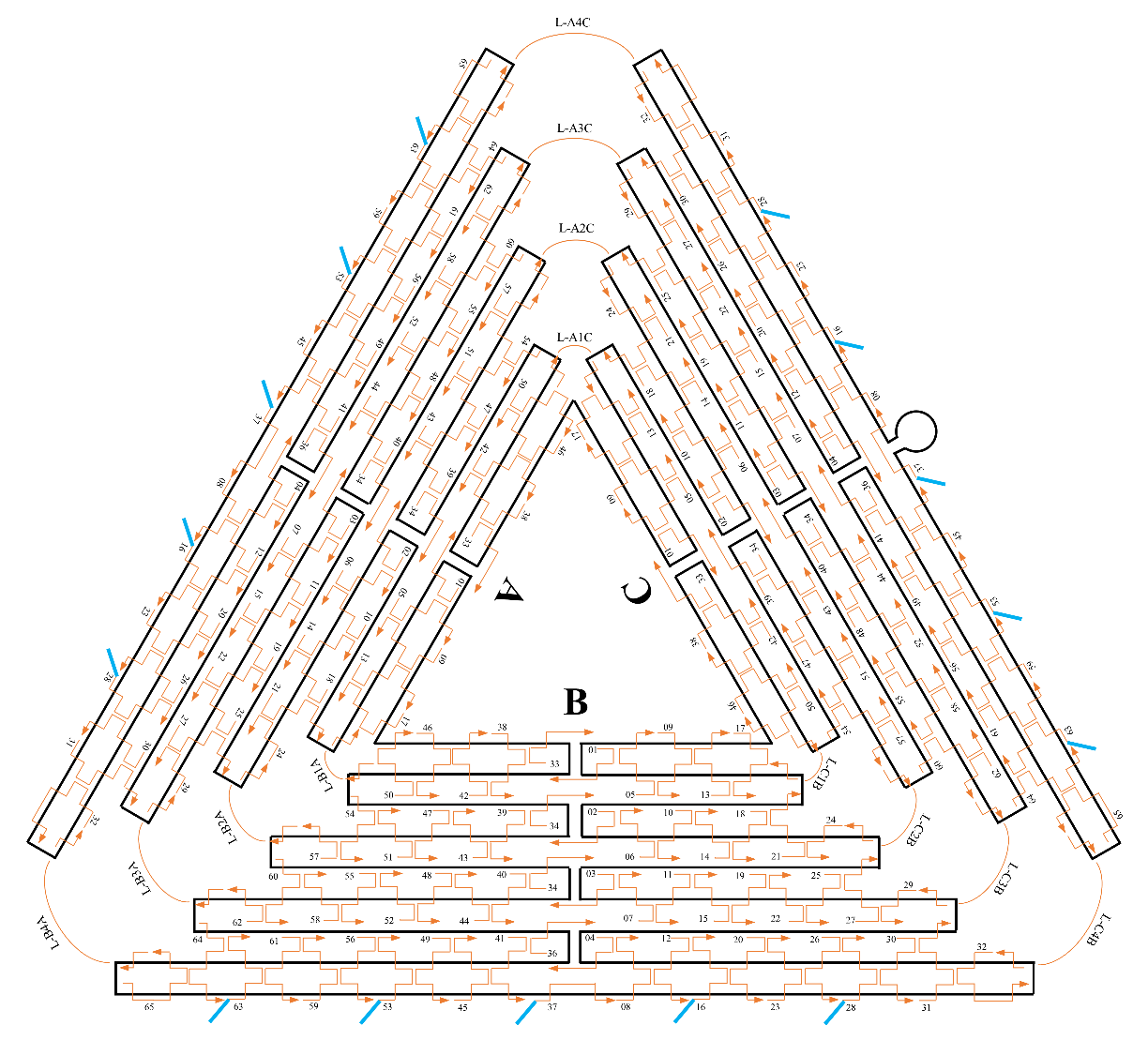


**Scheme S1**. The design of the AS1411 triangle DNA origami

1. AS1411 target strands for triangle DNA origami

AS1411-T,

TTTTTTTTTTTTTTTTTTGGTGGTGGTGGTTGTGGTGGTGGTGG

Green sequences represent DNA sequences of the aptamer AS1411 strands for targeting. Red sequences represent 18 nt hybridization capture strands

2. Capture strands for triangle DNA origami

A16-cap,

AAAAAAAAAAAAAAAA

GTCAGAGGGTAATTGATGGCAACATATAAAAGCGATTGAG

A28-cap,

AAAAAAAAAAAAAAAA

ATAAGAGCAAGAAACATGGCATGATTAAGACTCCGACTTG

A37-cap,

AAAAAAAAAAAAAAAA

AGAGAATAACATAAAAACAGGGAAGCGCATTA

A53-cap,

AAAAAAAAAAAAAAAA

TCCCAATCCAAATAAGATTACCGCGCCCAATAAATAATAT

A63-cap,

AAAAAAAAAAAAAAAA

ACGCTAACGAGCGTCTGGCGTTTTAGCGAACCCAACATGT

B16-cap,

AAAAAAAAAAAAAAAA

CGCCAAAAGGAATTACAGTCAGAAGCAAAGCGCAGGTCAG

B28-cap,

AAAAAAAAAAAAAAAA

GATAAAAACCAAAATATTAAACAGTTCAGAAATTAGAGCT

B37-cap,

AAAAAAAAAAAAAAAA

ACAGGTAGAAAGATTCATCAGTTGAGATTTAG

B53-cap,

AAAAAAAAAAAAAAAA

ACCAGTCAGGACGTTGGAACGGTGTACAGACCGAAACAAA

B63-cap,

AAAAAAAAAAAAAAAA

TGGTTTAATTTCAACTCGGATATTCATTACCCACGAAAGA

C16-cap,

AAAAAAAAAAAAAAAA

AGAATCAGAGCGGGAGATGGAAATACCTACATAACCCTTC

C28-cap,

AAAAAAAAAAAAAAAA

GAATCCTGAGAAGTGTATCGGCCTTGCTGGTACTTTAATG

C37-cap,

AAAAAAAAAAAAAAAA

CGAGAAAGGAAGGGAAGCGTACTATGGTTGCT

C53-cap,

AAAAAAAAAAAAAAAA

CTAAATCGGAACCCTAAGCAGGCGAAAATCCTTCGGCCAA

C63-cap,

AAAAAAAAAAAAAAAA

CGATGGCCCACTACGTATAGCCCGAGATAGGGATTGCGTT

3. Sequences of unmodified staple strands

A01, CGGGGTTTCCTCAAGAGAAGGATTTTGAATTA

A02, AGCGTCATGTCTCTGAATTTACCGACTACCTT

A03, TTCATAATCCCCTTATTAGCGTTTTTCTTACC

A04, ATGGTTTATGTCACAATCAATAGATATTAAAC

A05, TTTGATGATTAAGAGGCTGAGACTTGCTCAGTACCAGGCG

A06, CCGGAACCCAGAATGGAAAGCGCAACATGGCT

A07, AAAGACAACATTTTCGGTCATAGCCAAAATCA

A08, GACGGGAGAATTAACTCGGAATAAGTTTATTTCCAGCGCC

A09, GATAAGTGCCGTCGAGCTGAAACATGAAAGTATACAGGAG

A10, TGTACTGGAAATCCTCATTAAAGCAGAGCCAC

A11, CACCGGAAAGCGCGTTTTCATCGGAAGGGCGA

A12, CATTCAACAAACGCAAAGACACCAGAACACCCTGAACAAA

A13, TTTAACGGTTCGGAACCTATTATTAGGGTTGATATAAGTA

A14, CTCAGAGCATATTCACAAACAAATTAATAAGT

A15, GGAGGGAATTTAGCGTCAGACTGTCCGCCTCC

A16, GTCAGAGGGTAATTGATGGCAACATATAAAAGCGATTGAG

A17, TAGCCCGGAATAGGTGAATGCCCCCTGCCTATGGTCAGTG

A18, CCTTGAGTCAGACGATTGGCCTTGCGCCACCC

A19, TCAGAACCCAGAATCAAGTTTGCCGGTAAATA

A20, TTGACGGAAATACATACATAAAGGGCGCTAATATCAGAGA

A21, CAGAGCCAGGAGGTTGAGGCAGGTAACAGTGCCCG

A22, ATTAAAGGCCGTAATCAGTAGCGAGCCACCCT

A23, GATAACCCACAAGAATGTTAGCAAACGTAGAAAATTATTC

A24, GCCGCCAGCATTGACACCACCCTC

A25, AGAGCCGCACCATCGATAGCAGCATGAATTAT

A26, CACCGTCACCTTATTACGCAGTATTGAGTTAAGCCCAATA

A27, AGCCATTTAAACGTCACCAATGAACACCAGAACCA

A28, ATAAGAGCAAGAAACATGGCATGATTAAGACTCCGACTTG

A29, CCATTAGCAAGGCCGGGGGAATTA

A30, GAGCCAGCGAATACCCAAAAGAACATGAAATAGCAATAGC

A31, TATCTTACCGAAGCCCAAACGCAATAATAACGAAAATCACCAG

A32, CAGAAGGAAACCGAGGTTTTTAAGAAAAGTAAGCAGATAGCCG

A33, CCTTTTTTCATTTAACAATTTCATAGGATTAG

A34, TTTAACCTATCATAGGTCTGAGAGTTCCAGTA

A35, AGTATAAAATATGCGTTATACAAAGCCATCTT

A36, CAAGTACCTCATTCCAAGAACGGGAAATTCAT

A37, AGAGAATAACATAAAAACAGGGAAGCGCATTA

A38, AAAACAAAATTAATTAAATGGAAACAGTACATTAGTGAAT

A39, TTATCAAACCGGCTTAGGTTGGGTAAGCCTGT

A40, TTAGTATCGCCAACGCTCAACAGTCGGCTGTC

A41, TTTCCTTAGCACTCATCGAGAACAATAGCAGCCTTTACAG

A42, AGAGTCAAAAATCAATATATGTGATGAAACAAACATCAAG

A43, ACTAGAAATATATAACTATATGTACGCTGAGA

A44, TCAATAATAGGGCTTAATTGAGAATCATAATT

A45, AACGTCAAAAATGAAAAGCAAGCCGTTTTTATGAAACCAA

A46, GAGCAAAAGAAGATGAGTGAATAACCTTGCTTATAGCTTA

A47, GATTAAGAAATGCTGATGCAAATCAGAATAAA

A48, CACCGGAATCGCCATATTTAACAAAATTTACG

A49, AGCATGTATTTCATCGTAGGAATCAAACGATTTTTTGTTT

A50, ACATAGCGCTGTAAATCGTCGCTATTCATTTCAATTACCT

A51, GTTAAATACAATCGCAAGACAAAGCCTTGAAA

A52, CCCATCCTCGCCAACATGTAATTTAATAAGGC

A53, TCCCAATCCAAATAAGATTACCGCGCCCAATAAATAATAT

A54, TCCCTTAGAATAACGCGAGAAAACTTTTACCGACC

A55, GTGTGATAAGGCAGAGGCATTTTCAGTCCTGA

A56, ACAAGAAAGCAAGCAAATCAGATAACAGCCATATTATTTA

A57, GTTTGAAATTCAAATATATTTTAG

A58, AATAGATAGAGCCAGTAATAAGAGATTTAATG

A59, GCCAGTTACAAAATAATAGAAGGCTTATCCGGTTATCAAC

A60, TTCTGACCTAAAATATAAAGTACCGACTGCAGAAC

A61, GCGCCTGTTATTCTAAGAACGCGATTCCAGAGCCTAATTT

A62, TCAGCTAAAAAAGGTAAAGTAATT

A63, ACGCTAACGAGCGTCTGGCGTTTTAGCGAACCCAACATGT

A64, ACGACAATAAATCCCGACTTGCGGGAGATCCTGAATCTTACCA

A65, TGCTATTTTGCACCCAGCTACAATTTTGTTTTGAAGCCTTAAA

B01, TCATATGTGTAATCGTAAAACTAGTCATTTTC

B02, GTGAGAAAATGTGTAGGTAAAGATACAACTTT

B03, GGCATCAAATTTGGGGCGCGAGCTAGTTAAAG

B04, TTCGAGCTAAGACTTCAAATATCGGGAACGAG

B05, ACAGTCAAAGAGAATCGATGAACGACCCCGGTTGATAATC

B06, ATAGTAGTATGCAATGCCTGAGTAGGCCGGAG

B07, AACCAGACGTTTAGCTATATTTTCTTCTACTA

B08, GAATACCACATTCAACTTAAGAGGAAGCCCGATCAAAGCG

B09, AGAAAAGCCCCAAAAAGAGTCTGGAGCAAACAATCACCAT

B10, CAATATGACCCTCATATATTTTAAAGCATTAA

B11, CATCCAATAAATGGTCAATAACCTCGGAAGCA

B12, AACTCCAAGATTGCATCAAAAAGATAATGCAGATACATAA

B13, CGTTCTAGTCAGGTCATTGCCTGACAGGAAGATTGTATAA

B14, CAGGCAAGATAAAAATTTTTAGAATATTCAAC

B15, GATTAGAGATTAGATACATTTCGCAAATCATA

B16, CGCCAAAAGGAATTACAGTCAGAAGCAAAGCGCAGGTCAG

B17, GCAAATATTTAAATTGAGATCTACAAAGGCTACTGATAAA

B18, TTAATGCCTTATTTCAACGCAAGGGCAAAGAA

B19, TTAGCAAATAGATTTAGTTTGACCAGTACCTT

B20, TAATTGCTTTACCCTGACTATTATGAGGCATAGTAAGAGC

B21, ATAAAGCCTTTGCGGGAGAAGCCTGGAGAGGGTAG

B22, TAAGAGGTCAATTCTGCGAACGAGATTAAGCA

B23, AACACTATCATAACCCATCAAAAATCAGGTCTCCTTTTGA

B24, ATGACCCTGTAATACTTCAGAGCA

B25, TAAAGCTATATAACAGTTGATTCCCATTTTTG

B26, CGGATGGCACGAGAATGACCATAATCGTTTACCAGACGAC

B27, TAATTGCTTGGAAGTTTCATTCCAAATCGGTTGTA

B28, GATAAAAACCAAAATATTAAACAGTTCAGAAATTAGAGCT

B29, ACTAAAGTACGGTGTCGAATATAA

B30, TGCTGTAGATCCCCCTCAAATGCTGCGAGAGGCTTTTGCA

B31, AAAGAAGTTTTGCCAGCATAAATATTCATTGACTCAACATGTT

B32, AATACTGCGGAATCGTAGGGGGTAATAGTAAAATGTTTAGACT

B33, AGGGATAGCTCAGAGCCACCACCCCATGTCAA

B34, CAACAGTTTATGGGATTTTGCTAATCAAAAGG

B35, GCCGCTTTGCTGAGGCTTGCAGGGGAAAAGGT

B36, GCGCAGACTCCATGTTACTTAGCCCGTTTTAA

B37, ACAGGTAGAAAGATTCATCAGTTGAGATTTAG

B38, CCTCAGAACCGCCACCCAAGCCCAATAGGAACGTAAATGA

B39, ATTTTCTGTCAGCGGAGTGAGAATACCGATAT

B40, ATTCGGTCTGCGGGATCGTCACCCGAAATCCG

B41, CGACCTGCGGTCAATCATAAGGGAACGGAACAACATTATT

B42, AGACGTTACCATGTACCGTAACACCCCTCAGAACCGCCAC

B43, CACGCATAAGAAAGGAACAACTAAGTCTTTCC

B44, ATTGTGTCTCAGCAGCGAAAGACACCATCGCC

B45, TTAATAAAACGAACTAACCGAACTGACCAACTCCTGATAA

B46, AGGTTTAGTACCGCCATGAGTTTCGTCACCAGGATCTAAA

B47, GTTTTGTCAGGAATTGCGAATAATCCGACAAT

B48, GACAACAAGCATCGGAACGAGGGTGAGATTTG

B49, TATCATCGTTGAAAGAGGACAGATGGAAGAAAAATCTACG

B50, AGCGTAACTACAAACTACAACGCCTATCACCGTACTCAGG

B51, TAGTTGCGAATTTTTTCACGTTGATCATAGTT

B52, GTACAACGAGCAACGGCTACAGAGGATACCGA

B53, ACCAGTCAGGACGTTGGAACGGTGTACAGACCGAAACAAA

B54, ACAGACAGCCCAAATCTCCAAAAAAAAATTTCTTA

B55, AACAGCTTGCTTTGAGGACTAAAGCGATTATA

B56, CCAAGCGCAGGCGCATAGGCTGGCAGAACTGGCTCATTAT

B57, CGAGGTGAGGCTCCAAAAGGAGCC

B58, ACCCCCAGACTTTTTCATGAGGAACTTGCTTT

B59, ACCTTATGCGATTTTATGACCTTCATCAAGAGCATCTTTG

B60, CGGTTTATCAGGTTTCCATTAAACGGGAATACACT

B61, AAAACACTTAATCTTGACAAGAACTTAATCATTGTGAATT

B62, GGCAAAAGTAAAATACGTAATGCC

B63, TGGTTTAATTTCAACTCGGATATTCATTACCCACGAAAGA

B64, ACCAACCTAAAAAATCAACGTAACAAATAAATTGGGCTTGAGA

B65, CCTGACGAGAAACACCAGAACGAGTAGGCTGCTCATTCAGTGA

C01, TCGGGAGATATACAGTAACAGTACAAATAATT

C02, CCTGATTAAAGGAGCGGAATTATCTCGGCCTC

C03, GCAAATCACCTCAATCAATATCTGCAGGTCGA

C04, CGACCAGTACATTGGCAGATTCACCTGATTGC

C05, TGGCAATTTTTAACGTCAGATGAAAACAATAACGGATTCG

C06, AAGGAATTACAAAGAAACCACCAGTCAGATGA

C07, GGACATTCACCTCAAATATCAAACACAGTTGA

C08, TTGACGAGCACGTATACTGAAATGGATTATTTAATAAAAG

C09, CCTGATTGCTTTGAATTGCGTAGATTTTCAGGCATCAATA

C10, TAATCCTGATTATCATTTTGCGGAGAGGAAGG

C11, TTATCTAAAGCATCACCTTGCTGATGGCCAAC

C12, AGAGATAGTTTGACGCTCAATCGTACGTGCTTTCCTCGTT

C13, GATTATACACAGAAATAAAGAAATACCAAGTTACAAAATC

C14, TAGGAGCATAAAAGTTTGAGTAACATTGTTTG

C15, TGACCTGACAAATGAAAAATCTAAAATATCTT

C16, AGAATCAGAGCGGGAGATGGAAATACCTACATAACCCTTC

C17, GCGCAGAGGCGAATTAATTATTTGCACGTAAATTCTGAAT

C18, AATGGAAGCGAACGTTATTAATTTCTAACAAC

C19, TAATAGATCGCTGAGAGCCAGCAGAAGCGTAA

C20, GAATACGTAACAGGAAAAACGCTCCTAAACAGGAGGCCGA

C21, TCAATAGATATTAAATCCTTTGCCGGTTAGAACCT

C22, CAATATTTGCCTGCAACAGTGCCATAGAGCCG

C23, TTAAAGGGATTTTAGATACCGCCAGCCATTGCGGCACAGA

C24, ACAATTCGACAACTCGTAATACAT

C25, TTGAGGATGGTCAGTATTAACACCTTGAATGG

C26, CTATTAGTATATCCAGAACAATATCAGGAACGGTACGCCA

C27, CGCGAACTAAAACAGAGGTGAGGCTTAGAAGTATT

C28, GAATCCTGAGAAGTGTATCGGCCTTGCTGGTACTTTAATG

C29, ACCACCAGCAGAAGATGATAGCCC

C30, TAAAACATTAGAAGAACTCAAACTTTTTATAATCAGTGAG

C31, GCCACCGAGTAAAAGAACATCACTTGCCTGAGCGCCATTAAAA

C32, TCTTTGATTAGTAATAGTCTGTCCATCACGCAAATTAACCGTT

C33, CGCGTCTGATAGGAACGCCATCAACTTTTACA

C34, AGGAAGATGGGGACGACGACAGTAATCATATT

C35, CTCTAGAGCAAGCTTGCATGCCTGGTCAGTTG

C36, CCTTCACCGTGAGACGGGCAACAGCAGTCACA

C37, CGAGAAAGGAAGGGAAGCGTACTATGGTTGCT

C38, GCTCATTTTTTAACCAGCCTTCCTGTAGCCAGGCATCTGC

C39, CAGTTTGACGCACTCCAGCCAGCTAAACGACG

C40, GCCAGTGCGATCCCCGGGTACCGAGTTTTTCT

C41, TTTCACCAGCCTGGCCCTGAGAGAAAGCCGGCGAACGTGG

C42, GTAACCGTCTTTCATCAACATTAAAATTTTTGTTAAATCA

C43, ACGTTGTATTCCGGCACCGCTTCTGGCGCATC

C44, CCAGGGTGGCTCGAATTCGTAATCCAGTCACG

C45, TAGAGCTTGACGGGGAGTTGCAGCAAGCGGTCATTGGGCG

C46, GTTAAAATTCGCATTAATGTGAGCGAGTAACACACGTTGG

C47, TGTAGATGGGTGCCGGAAACCAGGAACGCCAG

C48, GGTTTTCCATGGTCATAGCTGTTTGAGAGGCG

C49, GTTTGCGTCACGCTGGTTTGCCCCAAGGGAGCCCCCGATT

C50, GGATAGGTACCCGTCGGATTCTCCTAAACGTTAATATTTT

C51, AGTTGGGTCAAAGCGCCATTCGCCCCGTAATG

C52, CGCGCGGGCCTGTGTGAAATTGTTGGCGATTA

C53, CTAAATCGGAACCCTAAGCAGGCGAAAATCCTTCGGCCAA

C54, CGGCGGATTGAATTCAGGCTGCGCAACGGGGGATG

C55, TGCTGCAAATCCGCTCACAATTCCCAGCTGCA

C56, TTAATGAAGTTTGATGGTGGTTCCGAGGTGCCGTAAAGCA

C57, TGGCGAAATGTTGGGAAGGGCGAT

C58, TGTCGTGCACACAACATACGAGCCACGCCAGC

C59, CAAGTTTTTTGGGGTCGAAATCGGCAAAATCCGGGAAACC

C60, TCTTCGCTATTGGAAGCATAAAGTGTATGCCCGCT

C61, TTCCAGTCCTTATAAATCAAAAGAGAACCATCACCCAAAT

C62, GCGCTCACAAGCCTGGGGTGCCTA

C63, CGATGGCCCACTACGTATAGCCCGAGATAGGGATTGCGTT

C64, AACTCACATTATTGAGTGTTGTTCCAGAAACCGTCTATCAGGG

C65, ACGTGGACTCCAACGTCAAAGGGCGAATTTGGAACAAGAGTCC

Link-A1C, TTAATTAATTTTTTACCATATCAAA

Link-A2C, TTAATTTCATCTTAGACTTTACAA

Link-A3C, CTGTCCAGACGTATACCGAACGA

Link-A4C, TCAAGATTAGTGTAGCAATACT

Link-B1A, TGTAGCATTCCTTTTATAAACAGTT

Link-B2A, TTTAATTGTATTTCCACCAGAGCC

Link-B3A, ACTACGAAGGCTTAGCACCATTA

Link-B4A, ATAAGGCTTGCAACAAAGTTAC

Link-C1B, GTGGGAACAAATTTCTATTTTTGAG

Link-C2B, CGGTGCGGGCCTTCCAAAAACATT

Link-C3B, ATGAGTGAGCTTTTAAATATGCA

Link-C4B, ACTATTAAAGAGGATAGCGTCC

Loop, GCGCTTAATGCGCCGCTACAGGGC
